# Cortical microinfarcts potentiate recurrent ischemic injury through NLRP3-dependent trained immunity

**DOI:** 10.1038/s41419-023-06414-7

**Published:** 2024-01-12

**Authors:** Yiwei Feng, Lishan Lin, Tengteng Wu, Yukun Feng, Fengyin Liang, Ge Li, Yongchao Li, Yalun Guan, Shuhua Liu, Yu Zhang, Guangqing Xu, Zhong Pei

**Affiliations:** 1 Department of Neurology, The First Affiliated Hospital, Sun Yat-sen University, Guangzhou, 510080 China; 2 Guangdong Provincial Key Laboratory of Diagnosis and Treatment of Major Neurological Diseases, National Key Clinical Department and Key Discipline of Neurology, Guangzhou, 510080 China; 3 Department of Neurology, Guangdong Provincial People’s Hospital (Guangdong Academy of Medical Sciences), Southern Medical University, No. 106 Zhongshan Road II, Guangzhou, 510080 China; 4 Department of Neurology, The First Affiliated Hospital of Guangzhou Medical University, Guangzhou, 510120 China; 5 Department of Neurology, Hainan General Hospital, 570311 Hainan, China; 6 Guangdong Provincial Key Laboratory of Laboratory Animals, Guangdong Laboratory Animals Monitoring Institute, Guangzhou, 510663 China; 7 Department of Rehabilitation Medicine, Guangdong Provincial People’s Hospital (Guangdong Academy of Medical Sciences), Southern Medical University, No. 106 Zhongshan Road II, Guangzhou, 510080 China

**Keywords:** Stroke, Neuroimmunology

## Abstract

Microinfarcts are common among the elderly and patients with microinfarcts are more vulnerable to another stroke. However, the impact of microinfarcts on recurrent stroke has yet to be fully understood. The purpose of this study was to explore the negative effects of microinfarcts on recurrent stroke. To achieve this, two-photon laser was used to induce microinfarcts, while photothrombotic stroke was induced on the opposite side. The results showed that microinfarcts led to trained immunity in microglia, which worsened the pro-inflammatory response and ischemic injury in the secondary photothrombotic stroke. Additionally, the study clarified the role of NLRP3 in microglial nuclei, indicating that it interacts with the MLL1 complex through NACHT domain and increases H3K4 methylation, which suggests that NLRP3 is critical in the formation of innate immune memory caused by microinfarcts. Furthermore, the knockout of NLRP3 in microglia alleviated the trained immunity and reduced the harmful effects of microinfarcts on recurrent stroke. This study emphasizes the detrimental effect of trained immunity on recurrent stroke and highlights the critical role of NLRP3 in mediating the formation of this memory, which may offer a potential therapeutic target for mitigating recurrent strokes.

## Facts

- Single microinfarct could worsen the subsequent stroke event through the long-term activation of trained immunity in microglia.
- H3K4 methylation by MLL1 is critical in initiating the microinfarct-induced trained immunity.
- NLRP3 is elevated in the microglial nuclei following microinfarct and subsequently activates trained immunity through MLL1-mediated methylation of H3K4.
- Knockout out of NLRP3 substantially alleviates the trained immunity and protects secondary stroke event.

## Introduction

Strokes are the leading cause of death worldwide. Cortical microinfarcts (CMIs) are frequently observed in older individuals. Approximately 48% of people over the age of 65 worldwide suffer from microinfarct [1]. The impact of CMIs on the brain is not limited to their specific locations but instead extends to a significant number of areas with a widespread distribution. It has been reported that patients who have experienced microinfarcts are at a heightened risk of suffering from additional strokes (15–42% over 5 years) [2]. Epidemiologically, recurrent strokes account for up to 40% of all strokes and are associated with high mortality and poor functional recovery [3]. Moreover, CMIs have been associated with worse outcomes in patients with acute ischemic stroke, including cognitive and motor function impairments [4]. However, the exact mechanism underlying this relationship is still unclear.

Inflammation and immune mediators play a critical role in stroke pathogenesis [5]. The inflammatory response in the brain is mainly mediated by microglia, which are highly plastic and phenotypically diverse cells that adapt to their microenvironment through different signaling pathways [6]. Recently, microglia have been reported to play a key role in the development of trained immunity in the brain through histone modifications after inflammatory stimulation [7]. Enhanced H3K4me1/3 modification can open the chromatin, resulting in easier transcription of the modified inflammatory gene, such as IL1β and TNF-α [8]. Trained microglia can exhibit more severe inflammatory responses and produce excessive pro-inflammatory cytokines once a secondary inflammatory stimulus occurs [9]. The deleterious role of trained immunity has been implicated in a mouse model of central nervous system (CNS) diseases, such as Alzheimer’s disease and stroke since a single dose of inflammatory stimulus can activate trained immunity [10]. These long-lasting (at least 6 months) trained microglia promote the deposition of amyloid β and aggravate stroke outcome over long time periods. Therefore, trained immunity in the CNS needs to be properly investigated.

The NLRP3 inflammasome is a multi-protein complex that is heavily involved in brain inflammation [11] as it mediates the cleavage of pro-inflammatory cytokines (e.g., IL-1β). High NLRP3 level has been observed in the ischemic brain of stroke animals, and NLRP3 inhibitor has been reported to alleviate ischemic brain damage in various stroke models [12, 13]. However, whether NLRP3 is involved in the development of trained immunity remains largely uninvestigated.

The present study explored whether CMIs can induce trained immunity and influence subsequent recurrent strokes. We also examined the role of NLRP3 in mediating CMI-induced trained immunity by promoting epigenetic reprogramming of MLL1 complex and subsequently increased H3K4 methylation. This revealed a novel therapeutic aspect to mitigate recurrent stroke consequences by targeting the NLRP3-MLL1 interaction.

## Materials and Methods

### Animals

C57BL/6J male mice (12-month-old, 20–25 g) were provided by the Sun Yat-sen University Medical Experimental Animal Center (Guangzhou, China). Animals were kept in a temperature- and humidity-controlled specific pathogen-free laboratory with a 12/12 h light/dark cycle. Animal experimental procedures were performed following the guidelines set by the Sun Yat-sen University Committee on the Care and Use of Animals.

Tamoxifen (T-143, Sigma-Aldrich, USA) was used to induce NLRP3 deletion in CX3CR1^CreER^ × NLRP3^fl/fl^ mice or CreER activation in *Cxc3r1*^CreER^ mice following a standard protocol [14]. Briefly, tamoxifen was first dissolved in corn oil at a concentration of 20 mg/mL by shaking overnight at 37 °C. It was then stored at 4 °C for the duration of injections. Tamoxifen was administered to animals via intraperitoneal injections once a day for five consecutive days at a dose of 75 mg/kg.

The genotype identification of *Cxc3r1*^CreER^ and ${{NLRP}3}_{{cxc}3r1}^{-/-}$ mice was performed as previously described [14] (Fig. S7). Briefly, mice tail was sampled, and DNA purification was performed using TIANamp Genomic DNA kit (DP304, Tiangen, China) according to the manufacture’s guide. And collected DNA sample was subjected to PCR analysis using TaKaRa Ex Taq® kit (RR001A, TaKaRa, Japan). The primer for *Cxc3r1*^CreER^ mice were listed as below: common: 5’-CCGCCAGACGCCCAGACTA; WT forward: 5’-AGCCGGAAGCCCAAGAGCATC; mutant forward: 5’-TGCTGCTGCCCGACAACCAC. The primer for ${{NLRP}3}_{{cxc}3r1}^{-/-}$ mice were listed as belows: FW: 5’-GAAGCTCCACAGGTCTCCCTT; RV: 5’-GGTTAGCCTGGGCTACACGAA.

And the PCR product were separated on 1-2.5% agarose gels and detected by Red Nucleic Acid Gel Stain (HY-K1007, MCE, USA).

### Microinfarct model

The microinfarct model was generated as previously described [15]. Briefly, mice were anesthetized with a combination of ketamine (0.12 mg/g, i.p.) and xylazine (0.01 mg/g, i.p.) and fixed on the stage of a two-photon microscope (Leica, DM6000, Wetzlar, Germany). Then, 0.1 mL of 0.3% 2000 KD FITC-dextran (FD20, Sigma-Aldrich, USA) was injected into the tail vein. A ROI measuring 15–20 µm in diameter and 10 µm/ms in velocity was selected as the target vessel for occlusion under 25× magnification water-immersion. Bleach mode with maximum laser power was used to damage the endothelium and bleach points located within the vessel lumen. Irradiation was initiated with an 800-nm laser and continued until a clot became apparent and red blood cell motion was stalled. Observation lasting for 1 h was required to evaluate the redistribution and stabilization of the downstream flow. Irradiation was repeated if any recanalization occurred. Mice models with hemorrhage or diffuse burns were discarded. MM102 (15 mg/kg, S7265, Selleck, USA) was intravenously injected immediately after microinfarction.

### MLL1-shRNA, NLRP3 mutant construction and rAAV/MG 1.2 construction and administration

In this study, shRNA targeting MLL1 (AATTATGGTCAAGTGAAGGCG) was used. To determine the role of different domains of NLRP3 in the interaction with MLL1, three truncated versions of NLRP3 including NLRP3 lacking LRR domain (NLRP3ΔLRR), NACHT (NLRP3ΔNACHT) and PYD (NLRP3ΔPYD) were amplified from genomic DNA by PCR and cloned into the pMXs-IRES-GFP by NEBuilder HiFi DNA Assembly Cloning kit (New England Biolabs, Ipswich, MA).

We further packaged these MLL1-shRNA and NLRP3 mutant plasmid into rAAV/MG1.2, created by Minmin Luo et al. [16]. This synthesis was constructed by GeneChem, Shanghai, China. Then, the rAAVs were stereoscopically injected into lateral ventricles of *Cxc3r1*^CreER^ mice.

Briefly, mice were anesthetized using 4% isoflurane and maintained on 2% isoflurane by a mask. Then, mice were placed on a stereotaxic injection instrument (68805, RWD, China). rAAVs were injected into the lateral ventricle (AP, −0.2 mm; ML, +1.5 mm; DV, −2.3 mm from bregma). 2 × 10^11^ genome copies were infused at a rate of 1 μl/min, after which the needle was left in place for 10 min to prevent backflow before withdrawal.

### Photothrombotic stroke model

After administering anesthesia using a combination of ketamine (0.12 mg/g, i.p.) and xylazine (0.01 mg/g, i.p.), mouse skulls were exposed by making a 2-mm incision 4 mm posterior and 3 mm left of the bregma for further investigation. Rose bengal dye (10 mg/mL, dissolved in saline) was injected into the tail vein at a dose of 0.03 mg/g body weight. The skull was then exposed to two-photon illumination with blue Hg light (450–500 nm) for 10 min focusing on the aperture incision. The skin was then sutured, and mice were kept on a warm heating pad to recover.

### Microglial isolation

The isolation of microglia was performed as previously described [17]. Briefly, mice were anesthetized with a combination of ketamine (0.12 mg/g, i.p.) and xylazine (0.01 mg/g, i.p.), and brains were placed in ice-cold phosphate-buffered saline (PBS) as quickly as possible. Brains were carefully minced into small fragments and digested with trypsin at 37 °C for 20 min. The digested tissue was then triturated and centrifuged at 400 g for 5 min. Centrifuged cells were collected and resuspended in 37% Percoll. The resuspended tissue was placed into 30% Percoll and 70% Percoll was added as the top layer. The tube was then centrifuged once more (700g) for 10 min. The cells between the 30 and 70% Percoll were collected and resuspended in Dulbecco’s Phosphate-Buffered Saline (DPBS)(~2 × 10^7^/mL). Cd11b^+^-positive magnetic isolation was then performed with a STEMCELL Easysep isolation kit (18970, EasySep™ Mouse CD11b Positive Selection Kit II, STEMCELL, Canada). Isolated cells were lysed in Radio Immunoprecipitation Assay (RIPA) buffer and subjected to western blot analysis.

### Nuclear and cytoplasmic protein extraction

Isolated Cd11b^+^ microglia were further processed to extract nuclear and cytoplasmic proteins. The isolation protocol was performed using NE-PER Nuclear and Cytoplasmic Extraction Reagents according to the manufacturer’s instructions (78833, ThermoFisher Scientific Inc., USA). Briefly, cells were resuspended in 50 μL of lysis buffer and incubated for 30 min on ice. Digested cells were lysed via ten strokes through a 26-gage needle and centrifuged for 10 min at 1000 *g*. The supernatant then contained the cytoplasm and mitochondria, while the pellet contained the nuclei.

### ChIP-qPCR

Chromatin precipitation was performed as previously described [18]. Briefly, isolated microglia were fixed in 0.75% formaldehyde for 10 min and quenched using glycine. Chromatin was then sonicated for four cycles of 10X (30 s ON, 30 s OFF) at 10% maximum power. Chromatin precipitation was performed using a rabbit anti-mouse H3K4me3 IgG Ab (5326, Cell Signaling Technology, USA). DNA was then quantified using qPCR with the following primer pairs: GAPDH, FW 59-ATCCAAGCGTGTAAGGGTCC-39, RV-59-ACTGAGATTGGCCCGATGG-39; IL6, FW 59-AGCTCTATCTCCCCTCCAGG-39; RV-59-ACAC-CCCTCCCTCACACAG-39; TNF-α, FW 59-CAGGCAGGTTCTCTTCCTCT-39, RV59-GCTTTCAGTGCTCATGGTGT-39.IL1b, FW-59-TGTGTGTCTTCCACTTTGTCCCAC-39; RV-59 CCTGACAATCGTTGTGCAGTTGATG-39; IL-10, FW 59- GGCGCTGTCATCGATTTCTC-39; RV-59- GCTCCACTGCCTTGCTCTTATTT-39. For all ChIP experiments, qPCR values were normalized as the percentage recovery of the input DNA.

### Western blot analysis

Mice were euthanized and perfused with ice-cold saline. The ischemic cortex was sampled and homogenized using ultrasonication in RIPA buffer. Western blot analysis was performed on protein samples of equal mass quantified using a Bicinchoninic Acid Assay (BCA) assay.

The following primary antibodies were used: anti-β-actin (1:1000, 4970, Cell Signaling Technology, USA), anti-IL-1β (1:1000, 31202, Cell Signaling Technology, USA), anti-IL-6 (1:1000, 12912, Cell Signaling Technology, USA), anti-H3K4me3 (1:1000; cell-signaling technology, USA), anti-H3K4me1 (1:1000, 5326, Cell Signaling Technology, USA), and anti-H3 (1:1000, 4499, Cell Signaling Technology, USA). Goat anti-rabbit HRP-linked antibody (1:1000, 7074, Cell Signaling Technology, USA) served as the secondary antibody.

### Immunofluorescence and in situ proximity ligation assay

Mice were euthanized, perfused with ice-cold saline, and fixed using 4% formaldehyde. After dehydration with 30% sucrose, coronal brain slices (20 µm thick) from the right parietal cortices were sectioned using a frozen microtome (CM1950, Leica, Germany) at 200-µm intervals.

For immunofluorescence experiments, slices were first permeabilized with 0.1% Triton X-100 in PBS for 15 min and then blocked with PBS containing 5% Bovine Serum Albumin (BSA) for 1 h. Primary antibodies diluted in blocking buffer were added to the sections and incubated at 4 °C overnight. The sections were then washed three times with PBS and incubated for 30 min at room temperature with the secondary antibody, then washed three more times with PBS. Next, the sections were mounted using DAPI mounting medium (F6057, Sigma-Aldrich, USA) for subsequent observations.

For proximity ligation assay experiments, cells were incubated for 1 h at 37 °C with the appropriate probes after primary antibodies were washed out and washed twice with PBS. Probes were then ligated for 30 min at 37 °C and washed twice in buffer A, then amplified for 100 min at 37 °C in the dark with polymerase (DUO92101, Sigma-Aldrich, USA).

The following primary antibodies were used in the experiment: anti-iba1 (1:500, ab5076, abcam, USA), anti-NLRP3 (1:200, MA5-16274, ThermoFisher Scientific Inc., USA), and anti-MLL1 (1:100, 14197, Cell-Signaling Technology, USA). The following secondary antibodies were used: donkey anti-rabbit IgG H&L (Alexa Fluor® 568) (1:1000; ab175470, abcam, USA), donkey anti-mouse IgG H&L (Alexa Fluor® 488) (1:1000; ab150105, abcam, USA), and donkey anti-goat IgG H&L (Alexa Fluor® 488) (1:1000; ab150129, abcam, USA).

### Nissl staining

Nissl staining was performed using serial frozen coronal sections 10 μm in thickness at 200-μm intervals from Posterior Parietal Cortex (PPC). The sections were hydrated in 1% toluidine blue at 37 °C overnight and washed using double distilled water. After soaking in dimethylbenzene for 5 s, sections were sealed in permount covered by a coverslip. Infarct volume (mm^3^) was used to measure the infarct size with Image J software (version 2.0.0-rc-68/1.52e).

### ELISA

Blood serum was obtained from the heart ventricle and sampled into coagulating mouse blood collection tubes (Vacutainer 367843, BD, USA) for 10 min at room temperature The blood was then centrifuged at 2000 g for 10 min to isolate the serum. Prior to cytokine measurements, the serum samples were diluted 1:2. After the blood collection, mice were perfused with ice-cold PBS and brain cortex was sampled in cell lysis buffer (EPX-99999-000, Invitrogen, USA), sonicated, and centrifuged at 25,000 g for 10 min. The supernatants were then collected for subsequent analysis.

The blood serum and brain homogenate samples were assayed using ELISA for IL-6 (M6000B, R&D Systems Inc., USA), IL-1β (MLB00C, R&D Systems Inc., USA), and TNF-α (MTA00B, R&D Systems Inc., USA) according to the manufacturer’s instructions.

### Sequence Alignment Assay

The protein structure of MLL1 (https://www.uniprot.org/uniprot/Q03164) was predicted using the open-source server trRosetta (https://yanglab.nankai.edu.cn/trRosetta/). The confidence score of the predicted structure model is: TM-score = 0.134. The protein structure of NLRP3 was based on PDB database (https://www.rcsb.org/structure/6NPY).

All the computational work were completed using the Rosetta 2020 program (Cambridge, USA). The docking method used was global docking, which allows the program to freely select the docking site. Parameters were set to default values unless otherwise specified.

The proteins to be docked were combined using Pymol and then subjected to global docking, generating 50,000 conformations for each protein complex. All generated conformations were evaluated using the InterfaceAnalyzer module in the Rosetta2020 program. Conformations with packstate ≥ 0.65 and dG_separated / dSASAx100 ≤ –1.5 were selected. (packstate: the degree of packing between protein interfaces (0 = no packing, 1 = perfect packing)). (dG_separated / dSASAx100: binding free energy per unit area). Finally, the conformation with the optimal binding free energy was analyzed as a potential protein binding mode. All docking results and scores are included in output_files.zip and pack_input_score.

### Statistical analysis

Data were analyzed using Graphpad Prism 9.5.0 and presented as mean ± standard deviation (SD). Experimental groups were randomized and investigators blinded to treatment conditions and mouse types for all outcome analyses. Randomization occurred pre-surgically using Graphpad’s random number generator. Sample sizes (*n*) are indicated in figure legends. One-way, two-way, or three-way ANOVA tests were utilized for data analyses based on the number of factors per group, with Tukey’s multiple comparisons to assess differences between individual groups. Significance was defined as *p* < 0.05. Regardless of test, results were equivalent in magnitude and statistically significant. Sample sizes were calculated to provide 95% power and α = 0.05.

## Results

### CMI aggravates secondary stroke

**Fig. 1:**
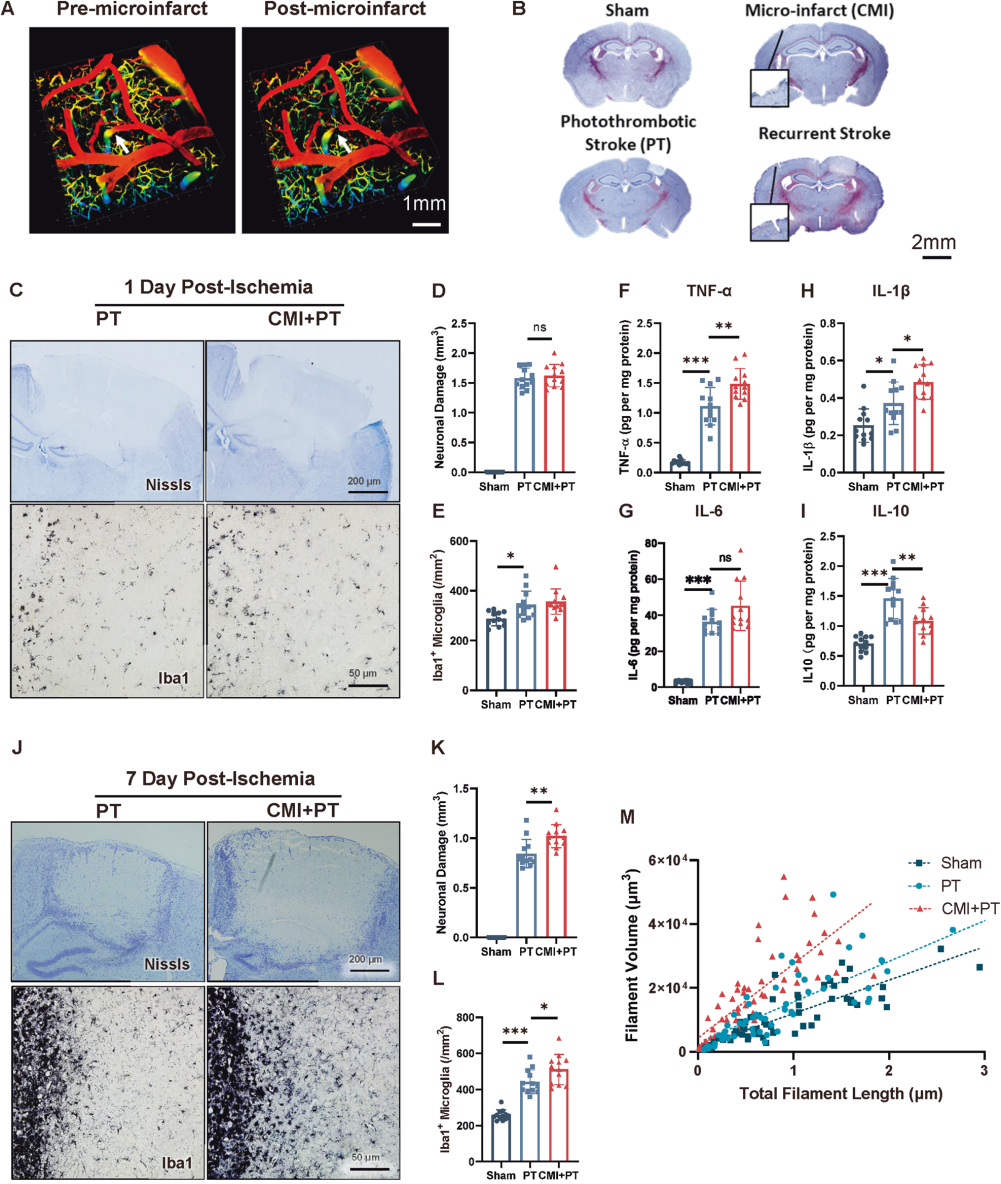
CMI initiates trained immunity and exacerbates secondary stroke in contralateral cortex. **A** Representative three-dimensional two-photon images showing CMI induction. Scale bar, 1 mm. **B** Representative Nissl staining showing the ischemic location and pathologies of CMI and PT. Scale bar, 2 mm. **C**–**E** Representative Nissl and Iba1 IHC staining and quantitative analysis of infarct size and microglial activation after one-day post-PT stroke. Scale bar, 200 μm for Nissl staining and 50 μm for Iba1 staining, *n* = 12 mice per group. One-way ANOVA *F*_2, 33_ = 468.2, *p* < 0.0001. PT vs. CMI+PT: *p* = 0.7213. (**D**) and *F*_2, 33_ = 7.497, *p* = 0.0021. PT vs. CMI+PT: *p* = 0.7471 (**E**) with Turkey’s correction. **F-I** Quantitative ELISA analysis of TNF-α (**F**), IL-6 (**G**), IL-1β (**H**), and IL-10 (**I**) expression in contralateral cortex 6 h after CMI. One-way ANOVA *F*_2, 33_ = 97.44, *p* < 0.0001. PT vs CMI+PT: *p* = 0.0013 (**F**); *F*_2, 33_ = 76.13, *p* < 0.0001. PT vs CMI+PT: *p* = 0.0511 (**G**); *F*_2, 33_ = 16.58, *p* < 0.0001. PT vs. CMI+PT: *p* = 0.0214 (**H**); *F*_2, 33_ = 28.71, *p* < 0.0001. PT vs. CMI+PT: *p* = 0.0020 (**I**). **J**–**M** Representative Nissl and Iba1 IHC staining and quantitative analysis of infarct size and microglial activation after seven-day post-PT stroke. Scale bar, 200 μm for Nissl staining and 50 μm for Iba1 staining, *n* = 12 mice per group. One-way ANOVA *F*_2, 33_ = 314.8, *p* < 0.0001. PT vs. CMI+PT: *p* = 0.0009 (**K**) and *F*_2, 33_ = 50.87, *p* < 0.0001. PT vs. CMI+PT: *p* = 0.0331 (**L**). Data are presented as mean ± standard deviation (SD), n.s., non-significant, *p < 0.05, **p < 0.01, and ***p < 0.001.

To investigate the influence of microinfarct on recurrent stroke, we first established a microinfarct mouse model using a two-photon microscope (Fig. 1A). The perforating artery with a diameter of 40 μm was lased and photothrombotic stroke was induced four weeks later (Fig. 1B). Acute and chronic pathologies of recurrent photothrombotic stroke were investigated. As the results showed, there was increased production of pro-inflammatory cytokines, including TNF-α, IL-6, and IL-1β, in the CMI+PT group compared to the PT group on the first day after ischemic stroke. However, no significant differences were observed in infarct volume, microglia activation, or astroglia activation between the CMI+PT and PT groups during this time period. (Fig. 1C–I, Fig. S1A–C). In the 7^th^ day post-stroke, the CMI+PT group displayed enhanced microglial activation characterized by shorter filament lengths and increased filament volumes compared to the PT group. (Fig. 1J, M). The increased activation of GFAP-positive astrocytes were also observed in CMI+PT group (Fig. S1D–F). As a result, the infarct volume is larger in the CMI+PT group (Fig. 1J–L). Inflammation was evident in the acute stage following stroke; however, brain edema was the primary factor affecting infarct size. In the chronic stage after stroke, the modulation of inflammatory responses was evident in mice with preceding CMI treatment and resulted in increased infarct volume. These findings suggest that microglia undergo long-term modification following CMI, resulting in exacerbated inflammatory responses and worsened stroke pathology during the secondary stroke event.

### CMI initiated microglial trained immunity in the contralateral cortex

**Fig. 2:**
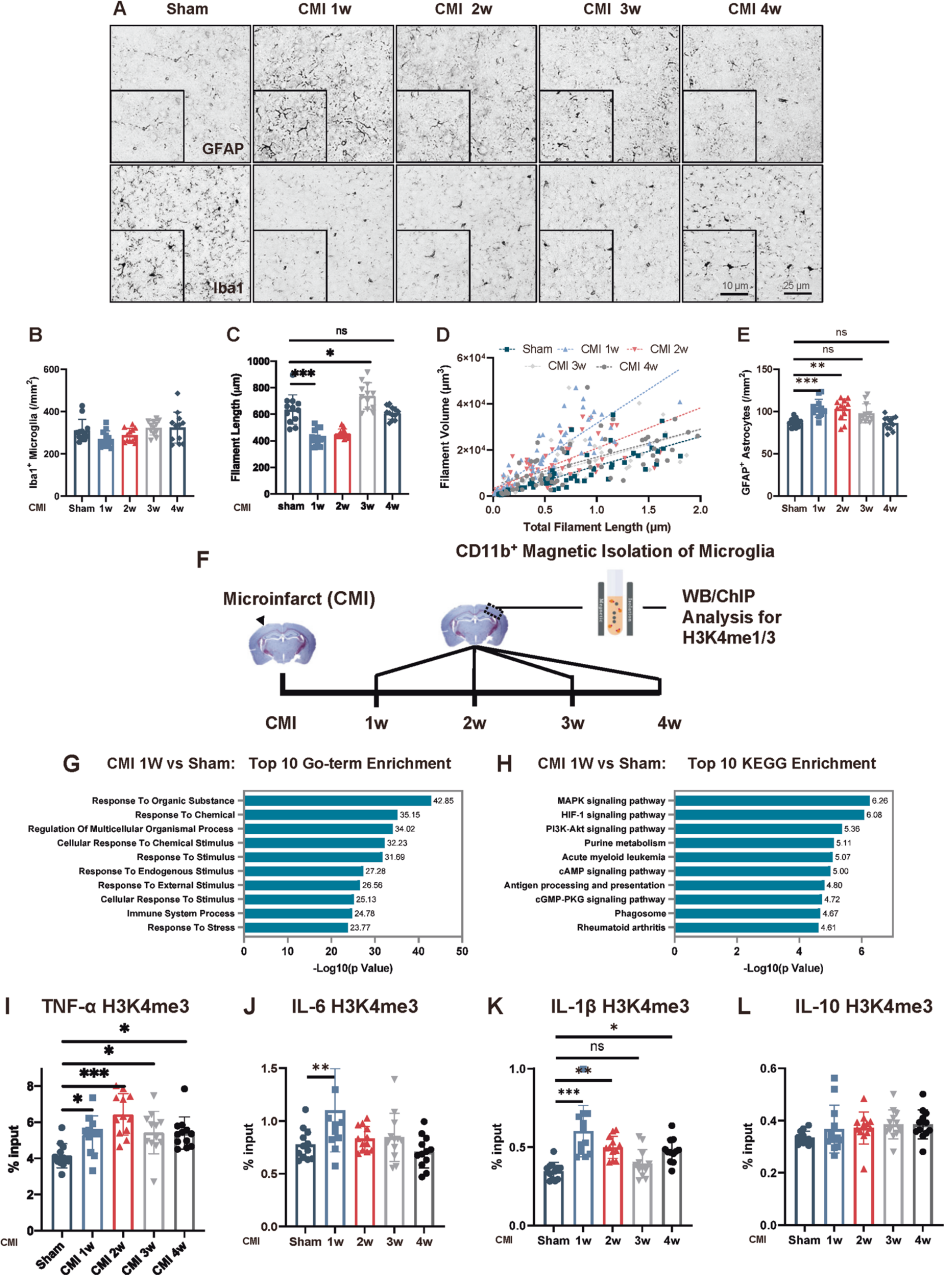
CMI initiated microglial trained immunity in the contralateral cortex. Representative GFAP and Iba1 IHC staining (**A**) and quantitative analysis for Iba-1 (**B–D**) and GFAP- (**E**) positive astrocytes around the contralateral cortex following CMI, *n* = 12 mice per group. One-way ANOVA *F*_4, 55_ = 2.808, *p* = 0.0342. Sham vs. 1w: *p* = 0.2748; Sham vs. 2w: *p* = 0.8075; Sham vs. 3w: *p* = 0.9621; Sham vs. 4w: *p* = 0.9515 (**B**); *F*_4, 55_ = 34.01, *p* < 0.0001; Sham vs. 1w: *p* < 0.001; Sham vs. 2w: *p* < 0.001; Sham vs. 3w: *p* = 0.0142; Sham vs. 4w: *p* = 0.9215 (**C**); and *F*_4, 55_ = 9.049, *p* < 0.0001; Sham vs. 1w: *p* = 0.0008; Sham vs. 2w: *p* = 0.0017; Sham vs. 3w: *p* = 0.0540; Sham vs 4w: *p* = 0.9999 (**E**) with Tukey’s correction. Scale bars, 25 μm. **F** Schematic diagram of experimental design. **G** Top 10 Gene Ontology (GO) enrichment terms from H3K4me3 ChIP-seq in contralateral microglia one week after CMI induction. **H** Top 10 Kyoto Encyclopedia of Genes and Genomes (KEGG) pathway enrichment components from H3K4me3 ChIP-seq in contralateral microglia one week after CMI induction. Quantitative analysis of H3K4me3 ChIP-qPCR on TNF-α (**I**), IL-6 (**J**), IL-1β (**K**), and IL-10 (**L**) promoters in contralateral microglia after CMI induction, *n* = 12 mice per group. One-way ANOVA. *F*_4, 55_ = 7.884, *p* < 0.0001; Sham vs CMI1w: *p* = 0.0434; Sham vs. CMI 2w: *p* < 0.0001; Sham vs. CMI 3w: *p* = 0.0218; Sham vs. CMI 4w: *p* = 0.0256 (**I**); *F*_4, 55_ = 5.031, *p* = 0.0016; Sham vs. CMI1w: *p* = 0.0091; Sham vs. CMI 2w: *p* = 0.9744; Sham vs. CMI 3w: *p* = 0.9477; Sham vs. CMI 4w: *p* = 0.9488 (**J**); *F*_4, 55_ = 11.98, *p* < 0.0001; Sham vs. CMI1w: *p* < 0.0001; Sham vs. CMI 2w: *p* = 0.0037; Sham vs. CMI 3w: *p* = 0.7670; Sham vs. CMI 4w: *p* = 0.0183 (**K**); and *F*_4, 55_ = 1.38, *p* = 0.253; Sham vs CMI1w: *p* = 0.6866; Sham vs. CMI 2w: *p* = 0.5709; Sham vs CMI 3w: *p* = 0.2671; Sham vs. CMI 4w: *p* = 0.2671 (**L**) with Tukey’s correction. Data are presented as mean ± standard deviation (SD), n.s., non-significant, *p < 0.05, **p < 0.01, and ***p < 0.001.

The increased inflammatory responses and deteriorated pathology of PT stroke following CMI indicated that trained immunity might be involved. To investigate this, we first examined the morphology of microglia and astrocytes in the contralateral cortex after CMI (Fig. 2A). Microglial morphology was significantly changed, as evidenced by the decreased microglial filament length and increased filament volume (Fig. 2B–D). Additionally, the number of GFAP-positive astrocytes was robustly increased in the first 2 weeks post CMI (Fig. 2A, E). This activation of glial cells gradually returned to normal levels four weeks later (Fig. 2B–E), suggesting the activation of glial cells in the contralateral cortex after CMI and we speculated that this activation might prime the trained immunity in the microglia and influenced the subsequent stroke event.

Trained immunity in the brain is driven by epigenetic changes in microglia though H3 methylation. Since the activation of microglia was prominent one week post CMI, we isolated CD11b-positive microglia in the contralateral CMI side and performed ChIP-seq analysis (Fig. 2F). The gene ontology (GO) analysis revealed a significant enrichment of biological processes related to external stimulation, while Kyoto Encyclopedia of Genes and Genomes (KEGG) analysis showed a significant enrichment of pathways associated with inflammatory responses, such as HIF-α, which is typically increased in LPS-induced trained immunity [7] (Fig. 2G, H). Moreover, the expression levels of H3K4me1 and H3K4me3 increased after exposure to CMI and remained elevated for a duration of four weeks (Fig. S1G–I). Therefore, we also examined the H3K4me3 modification on the promoters of TNF-α, IL-6, IL-1β, and IL-10 and found a significant increase in TNF-α, IL-6, IL-1β following CMI. Notably, this H3K4me3 modification persisted for at least four weeks, indicating that microglia were epigenetically modified following CMI and retained their altered state at least for four weeks (Fig. 2I–L).

### H3 methylation inhibition abolished the detrimental effect of CMI on recurrent PT stroke

**Fig. 3:**
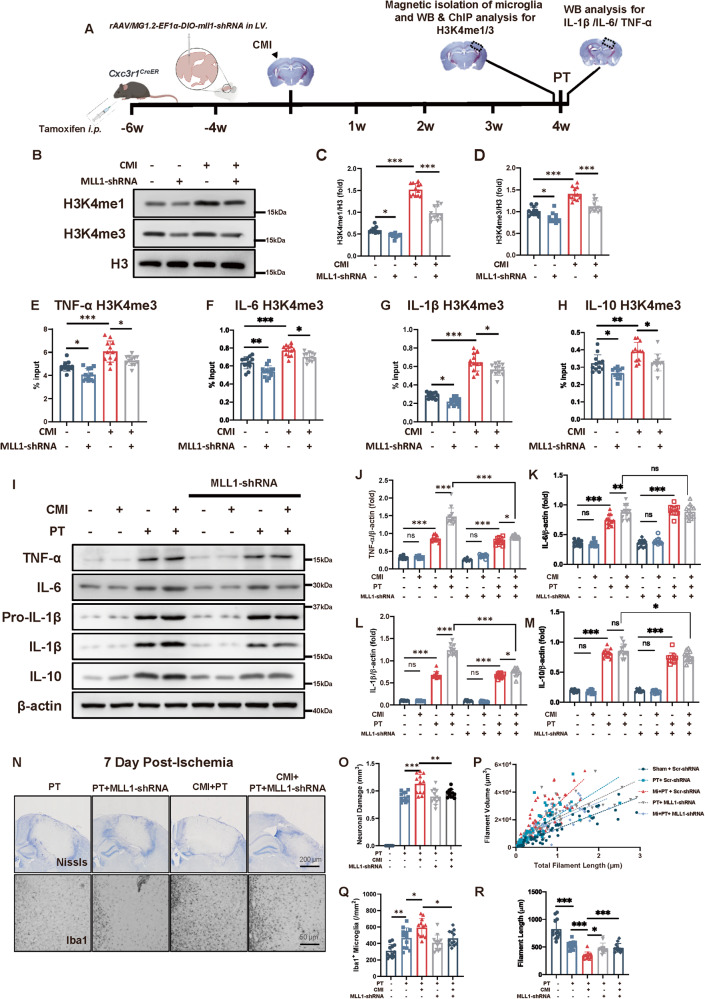
Inhibition of H3 methylation abolished the detrimental effect of CMI on recurrent PT stroke. **A** Schematic diagram of experimental design. **B**–**D** Immunoblots and quantitative analysis of H3K4me1/3 showing microglial H3K4me1/3 expression in contralateral mouse cortex subjected to CMI and MLL1-shRNA treatment, *n* = 12 mice per group. Two-way ANOVA, for interaction: *p* < 0.0001. So, t-tests were used to detect the difference between groups. Sham vs. CMI: *p* < 0.0001; CMI vs. CMI+ MLL1-shRNA: *p* < 0.0001 (**C**) For interaction: *p* = 0.0774. For MLL1-shRNA factor: *p* < 0.0001. For CMI factor: *p* < 0.0001. Sham vs. CMI: *p* < 0.0001; CMI vs. CMI+ MLL1-shRNA: *p* < 0.0001 (**D**) with Turkey’s correction. Quantitative analysis of H3K4me3 ChIP-qPCR on TNF-α (**E**), IL-6 (**F**), IL-1β (**G**), and IL-10 (**H**) promoters in contralateral microglia 4 weeks after CMI and MLL1-shRNA treatment, *n* = 12 mice per group. Two-way ANOVA, for interaction *p* = 0.8532. For MLL1-shRNA factor, *p* < 0.0001. CMI vs. CMI+ MLL1-shRNA: *p* = 0.0129 (**E**); For interaction *p* = 0.3404. For MLL1-shRNA factor, *p* < 0.0001. CMI vs. CMI+MLL1-shRNA: *p* = 0.0238 (**F**); For interaction *p* = 0.7885. For MLL1-shRNA factor, *p* = 0.0002. CMI vs. CMI+ MLL1-shRNA: *p* = 0.0165 (**G**); For interaction *p* = 0.2121. For MLL1-shRNA factor, *p* = 0.0645. CMI vs. CMI+MLL1-shRNA: *p* = 0.9701 (**H**) with Tukey’s correction. **I**–**M** Immunoblots and quantitative analysis of TNF-α (**J**), IL-6 (**K**), IL-1β (**L**), and IL-10 (**M**) showing pro-inflammatory cytokine expression in peri-infarct region 12 h after PT stroke, *n* = 12 mice per group. Three-way ANOVA. For interaction, *p* < 0.0001. So, t-tests were used to detect the difference between groups. PT vs. CMI+PT: *p* < 0.0001; CMI+PT vs. CMI+PT+MLL1-shRNA: *p* < 0.0001 (**J**); For interaction, *p* = 0.0026. So, t-tests were used to detect the difference between groups. PT vs. CMI+PT: *p* = 0.0056; CMI+PT vs. CMI+PT+MLL1-shRNA: *p* = 0.8656 (**K**); For interaction, *p* < 0.0001. So, t-tests were used to detect the difference between groups. PT vs. CMI+PT: *p* < 0.0001; CMI+PT vs. CMI+PT+MLL1-shRNA: *p* < 0.0001 (**L)** For interaction, *p* = 0.7475. PT vs. CMI+PT: *p* = 0.7733; CMI+PT vs. CMI+PT+MLL1-shRNA: *p* = 0.0188 (**M**) with Tukey’s correction. Representative Nissl and Iba1 IHC staining (**N**) and quantitative analysis of infarct size and microglial activation (**O**–**R**) showing PT stroke exacerbated by CMI was potentially mitigated by H3 methylation inhibition, *n* = 12 mice per group. Two-way ANOVA. For interaction, *p* = 0.0205. So, t-tests were used to detect the difference between groups. CMI+PT vs. CMI+PT+MLL1-shRNA: *p* = 0.0046 (**O**); For interaction, *p* = 0.2487. CMI+PT vs. CMI+PT+ MLL1-shRNA: *p* = 0.0228 (**Q**); For interaction, *p* < 0.0001. So, t-tests were used to detect the difference between groups. CMI+PT vs. CMI+PT++ MLL1-shRNA: *p* = 0.0001 (**R**) with Tukey’s correction. Scale bar, 200 μm for Nissl staining and 50 μm for Iba1 staining. Data are presented as mean ± standard deviation (SD), *p < 0.05, **p < 0.01, ***p < 0.001, n.s., non-significant.

The increased H3 methylation and its binding on the promoter of pro-inflammatory cytokines after CMI suggested the participation of trained immunity in the exacerbation of recurrent stroke. To verify that trained immunity was involved, we knocked down the MLL1, a crucial protein for MLL1 complex to induce H3 methylation [19], using MLL1-shRNA which was packed into AAV-MG1.2. And we injected the rAAVs into the lateral ventricle of the *Cxc3r1*^CreER^ mice. Utilizing this specialized method, pioneered by Minmin Luo et al., we achieved precise transduction of microglia in vivo [16] (Fig. S2A). Microglial MLL1 was inhibited by rAAVs injection when CMI was induced (Fig. 3A and Fig. S2B, C). Interestingly, microglial H3K4me1 and H3K4me3 levels were significantly inhibited by MLL1-shRNA in CMI mice (Fig. 3B–D), which were accompanied by the abolished H3K4me3 modification on TNF-α, IL-6, IL-1β, and IL-10 promoters (Fig. 3E–H).

Since trained immunity promoted the increased inflammatory response, TNF-α, IL-6, IL-1β, and IL-10 expression levels after CMI were detected. The results showed that CMI significantly increased TNF-α, IL-6 and IL-1β production 6 h after recurrent PT stroke, whereas MLL1 knockdown abolished this increase (Fig. 3I–M). Accordingly, the infarct size and microglial number around the infarct region were also inhibited by MLL1 knockdown seven days post PT stroke (Fig. 3N, O, and Q). And the microglia exhibited increased filament length, alleviated filament volume by MLL1 knockdown (Fig. 3P, R). The infarct size and number of microglia around the para infarct region one day post PT stroke did not show significant differences (Fig. S3A, B). TNF-α, IL-6, IL-1β, and IL-10 were also measured in peripheral blood samples to examine the possible effect of peripheral inflammatory response on the recurrent stroke. The results showed that PT stroke with preceding CMI did not influence the peripheral inflammatory cytokine production and H3 methylation inhibition did not alter the inflammatory status in the peripheral (Fig. S3C–F). Similar results were also validated by MM102 administration, which was a high affinity peptidomimetic inhibitor of the WDR5/MLL1 complex (Figs. S4, S5).

### CMI primes the microglial-trained immunity through NLRP3 in contralateral cortex

**Fig. 4:**
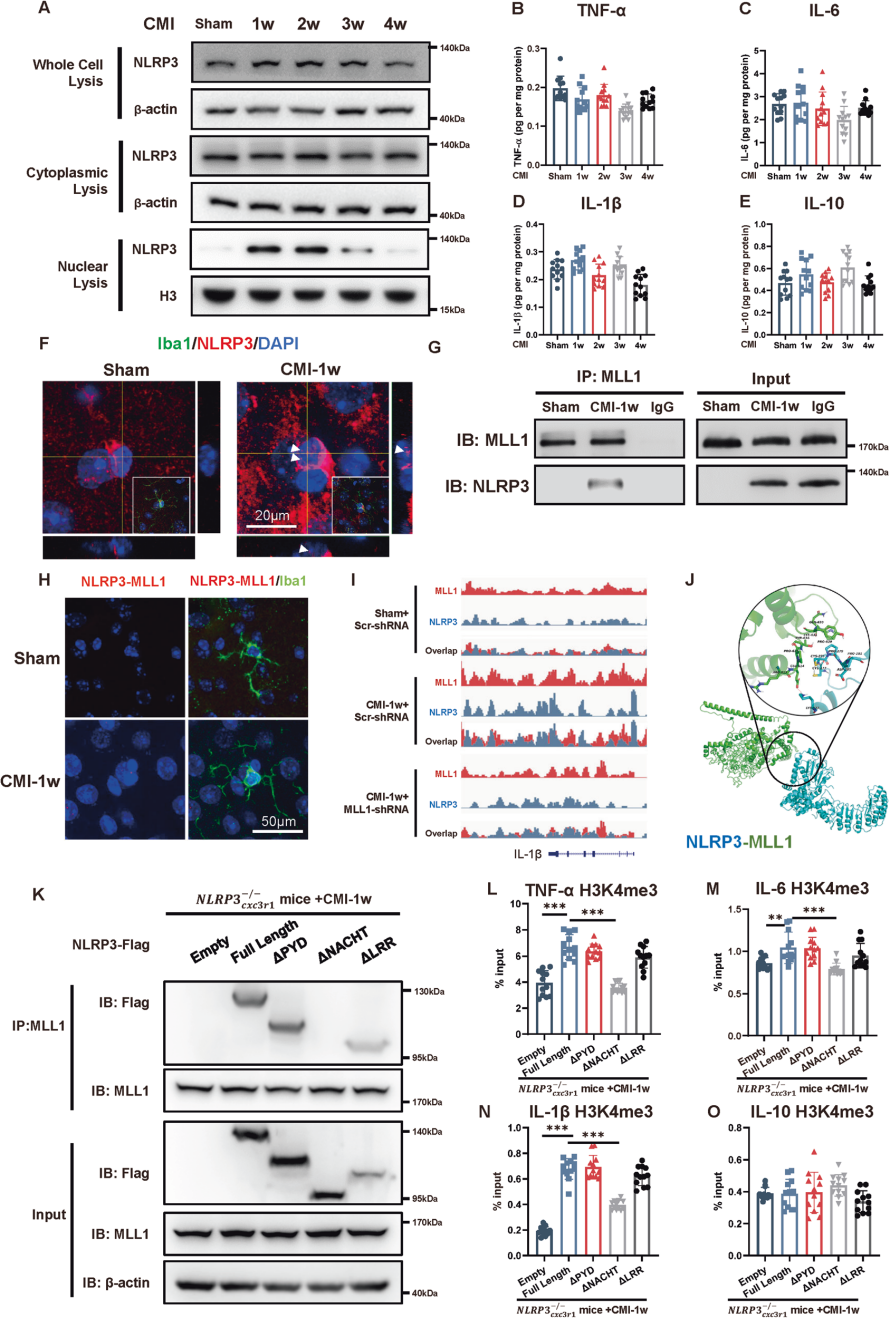
CMI-induced interaction of NLRP3 with MLL1 in contralateral cortex. **A** Immunoblots of NLRP3 expression in microglial whole-cell, cytoplasmic, and nuclear lysis after CMI, *n* = 12 mice per group. Quantitative ELISA analysis of TNF-α (**B**), IL-6 (**C**), IL-1β (**D**) and IL-10 (**E**) expression in the contralateral side of CMI showing that CMI did not influence the inflammatory cytokines expression in the chronic phase of CMI. (*n* = 12 mice per group). One-way ANOVA *F*_4, 55_ = 8.251, *p* < 0.0001. Sham vs. CMI1w: *p* = 0.0867; Sham vs. CMI 2w: *p* = 0.9064; Sham vs. CMI 3w: *p* < 0.0001; Sham vs. CMI 4w: *p* = 0.0331 (**B**); F _4, 55_ = 3.113, *p* = 0.0222. Sham vs. CMI1w: *p* = 0.9992; Sham vs. CMI 2w: *p* = 0.9211; Sham vs. CMI 3w: *p* = 0.0390; Sham vs. CMI 4w: *p* = 0.9554 (**C**); F_4, 55_ = 10.62, *p* < 0.0001. Sham vs. CMI1w: *p* = 0.2167; Sham vs. CMI 2w: *p* = 0.5763; Sham vs. CMI 3w: *p* = 0.9177; Sham vs. CMI 4w: *p* = 0.0027 (**D**); F_4, 55_ = 4.098, *p* = 0.0056. Sham vs. CMI1w: *p* = 0.4233; Sham vs. CMI 2w: *p* > 0.9999; Sham vs. CMI 3w: *p* = 0.0266; Sham vs. CMI 4w: *p* = 0.9984 (**E**) with Tukey’s correction. **F** Representative immunofluorescence images of NLRP3 (red) expression in microglia (iba1, green) one week after CMI. Scale bars, 20 μm. **G** Immunoprecipitation and immunoblot analysis of MLL1 and NLRP3 in microglial lysates from mice subjected to sham and CMI. Lysates were immunoprecipitated by anti-MLL1 (IP) or immunoglobulin control (IgG) or directly subjected to immunoblot (Input), *n* = 3 mice per group. **H** Proximity ligation assay of microglial (iba1, green) NLRP3 and MLL1 (red dots) in sham and one-week post-CMI mice. Scale bars, 20 μm. **I** ChIP-seq analysis of NLRP3 and MLL1 binding to IL-1β in one-week post-CMI mice. **J** Representative images of sequence alignments showing interaction of NLRP3 (blue) with MLL1 (green). **K** Immunprecipitation and immunoblot analysis of MLL1 and Flag in microglial lysates from Cxc3r1^CreER^ mice transfected with 3xFlag-NLRP3 or truncated mutants (ΔPYD, ΔNACHT, ΔLRR) and subjected to CMI surgery. Lysates were immunoprecipitated by anti-MLL1 (IP) or immunoglobulin control (IgG) or directly subjected to immunoblot (Input), *n* = 3 mice per group. Quantitative analysis of H3K4me3 ChIP-qPCR on TNF-α (**L**), IL-6 (**M**), IL-1β (**N**), and IL-10 (**O**) promoters in contralateral microglia one week after CMI. Cxc3r1^CreER^ mice transfected with 3xFlag-NLRP3 or truncated mutants (ΔPYD, ΔNACHT, ΔLRR) and subjected to CMI surgery. One-way ANOVA, *F*_4, 55_ = 42.25, *p* < 0.0001;Empty vs Full Length, *p* < 0.0001; Full Length vs ΔPYD: *p* = 0.7675; Full length vs ΔNACHT, *p* < 0.0001; Full Length vs ΔLRR: *p* = 0.1030 (**L**); *F*_4, 55_ = 8.732, *p* < 0.0001; Empty vs. Full Length, *p* = 0.0077; Full Length vs ΔPYD: *p* = 0.9998; Full length vs ΔNACHT, *p* = 0.0001; Full Length vs. ΔLRR: *p* = 0.3921(**M**); *F*_4, 55_ = 122.5, *p* < 0.0001;Empty vs. Full Length, *p* < 0.0001; Full Length vs ΔPYD: *p* = 0.9868; Full length vs. ΔNACHT, *p* < 0.0001; Full Length vs. ΔLRR: *p* = 0.2638 (**N**); *F*_4, 55_ = 2.246, *p* = 0.0758; Empty vs Full Length, *p* > 0.9999; Full Length vs ΔPYD: *p* = 0.9988; Full length vs ΔNACHT, *p* = 0.5413; Full Length vs. ΔLRR: *p* = 0.6035(**O**). *n* = 12 mice per group. Data are presented as mean ± standard deviation (SD). *p < 0.05, **p < 0.01, ***p < 0.001, n.s., non-significant.

NLRP3, which is critical in mediating the cleavage of the IL-1β, has recently been reported to mediate trained immunity [20]. To investigate the effect of NLRP3 in trained immunity on recurrent stroke, the expression of NLRP3 was first measured in microglia isolated from the cortex contralateral to the CMI. The NLRP3 expression robustly increased within the first 2 weeks and gradually returned to the baseline level over the next 2 weeks (Fig. 4A and Fig. S6A–C). However, IL-1β production and other proinflammatory cytokines remained at the baseline level (Fig. 4B–E), indicating that NLRP3 functioned in an inflammasome-independent manner. The localization of NLRP3 in isolated microglia was therefore examined. The results showed that NLRP3 expression was significantly higher in the nuclei within the first two weeks after CMI, whereas its expression in the cytoplasm remained unchanged (Fig. 4A). The nuclear localization of NLRP3 was further confirmed by immunofluorescent staining in the contralateral cortex in mice 1 week post CMI (Fig. 4F). H3 was majorly methylated by the MLL1 complex, which is important in initiating trained immunity. Therefore, the interaction between NLRP3 and MLL1 was examined using immunoprecipitation on microglia isolated from the non-ischemic cortex one week after CMI. The results indicated that NLRP3 interacted with MLL1 in the microglia of CMI mice one week after the surgery while not in the sham group (Fig. 4G). The interaction was further confirmed in microglial nuclei using a proximity ligation assay (Fig. 4H). The ChIP-seq analysis further verified that the MLL1 peak found in the il1b showed a significant increased overlap with NLRP3 peak after CMI treatment (Fig. 4I).

To identify potential interaction sites between MLL1 and NLRP3, sequence alignment analysis was first performed (Fig. 4J). The NACHT domain of NLRP3, specifically Cys277-Pro281 residues, emerged as an ideal docking site based on a favorable binding free energy of -9.884 kcal/mol. Therefore, we constructed mutant NLRP3 with deletion of one of the structural domains (LRR, NACHT, or PYD), packaged into AAV-MG1.2, and transduced into microglia though stereotactic injection into lateral ventricle of ${{NLRP}3}_{{cxc}3r1}^{-/-}$ mice. One week after CMI, mice were sacrificed and subjected to immunoprecipitation assay. The result showed that the NACHT domain of NLRP3 was required for interacting with MLL1 (Fig. 4K). More importantly, the NACHT-deleted NLRP3 variant failed to induce the H3 modification on inflammatory genes (Fig. 4L–O). This underscores the importance of the NACHT domain for NLRP3 to interact with MLL1 and promote H3 modifications on inflammatory genes.

Collectively, contralateral nuclear NLRP3 in the microglia was elevated in the chronic stage of microinfarct and interacted with MLL1, which mediate the activation of trained immunity.

### Microglial knockout of NLRP3 mitigated H3 methylation and attenuated CMI-induced detrimental effects on recurrent stroke

**Fig. 5:**
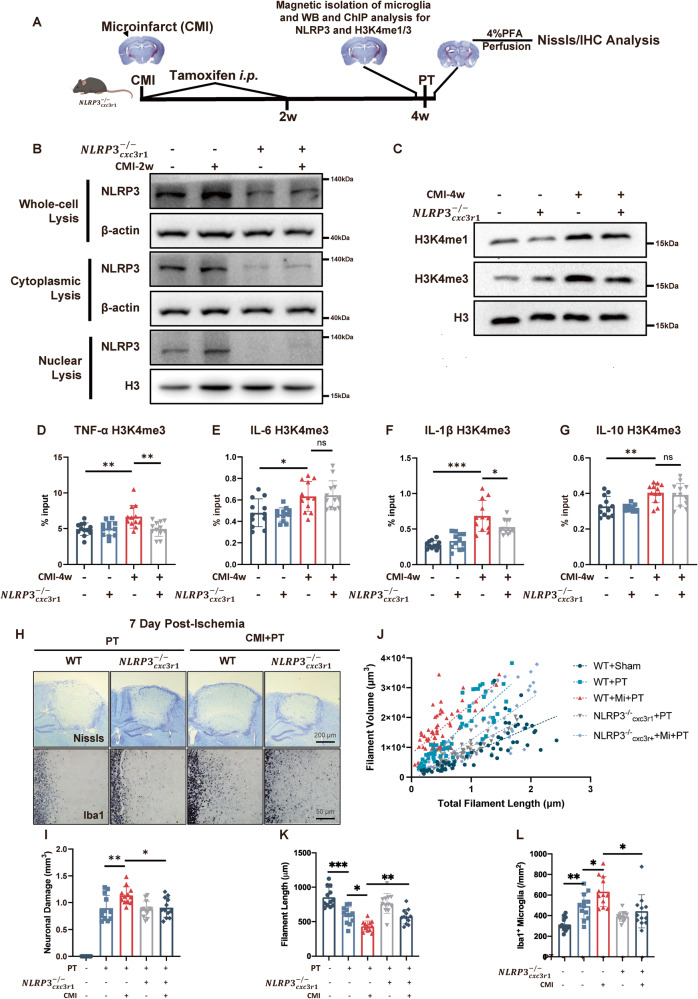
Knockout of microglial NLRP3 mitigated MLL1, reduced H3 methylation, and attenuated CMI-induced detrimental effects on recurrent stroke. **A** Schematic diagram of experimental design. **B** Immunoblot of microglial NLRP3 expression in WT and ${{NLRP}3}_{{cxc}3r1}^{-/-}$ mice four weeks after CMI induction, *n* = 12 mice per group. **C** Immunoblot of microglial H3K4me1/3 expression in WT and ${{NLRP}3}_{{cxc}3r1}^{-/-}$ mice four weeks after CMI induction, *n* = 12 mice per group. Quantitative analysis of H3K4me3 ChIP-qPCR on TNF-α (**D**), IL-6 (**E**), IL-1β (**F**), and IL-10 (**G**) promoters among contralateral microglia four weeks after CMI induction in WT and ${{NLRP}3}_{{cxc}3r1}^{-/-}$ mice, *n* = 12 mice per group. Two-way ANOVA, for interaction: *p* = 0.0110. So, t-tests were used to detect the difference between groups. CMI vs. CMI+${{NLRP}3}_{{cxc}3r1}^{-/-}$: *p* = 0.0053 (**D**); For interaction: *p* = 0.7059. CMI vs. CMI+${{NLRP}3}_{{cxc}3r1}^{-/-}$: *p* = 0.9965 (**E**); For interaction: *p* = 0.0106. So, t-tests were used to detect the difference between groups. CMI vs. CMI+${{NLRP}3}_{{cxc}3r1}^{-/-}$: *p* = 0.0341 (**F**); For interaction: *p* = 0.0003. So, t-tests were used to detect the difference between groups. CMI vs. CMI+${{NLRP}3}_{{cxc}3r1}^{-/-}$: *p* = 0.6246 (**G**) with Tukey’s correction. Representative Nissl and Iba1 IHC staining (**H**) and quantitative analysis of infarct size and microglial activation (**I**–**L**) showing that NLRP3 blockade in microglia after CMI abolished the exacerbated infarct size and microglial activation by CMI, *n* = 12 mice per group. Two-way ANOVA, for interaction: *p* = 0.6293. CMI vs. CMI+${{NLRP}3}_{{cxc}3r1}^{-/-}$: *p* = 0.0231 (**I**), for interaction: *p* = 0.2277. CMI vs CMI+${{NLRP}3}_{{cxc}3r1}^{-/-}$: *p* = 0.0034 (**K**), for interaction: *p* = 0.0617. CMI vs. CMI+${{NLRP}3}_{{cxc}3r1}^{-/-}$: *p* = 0.0229 (**L**) with Tukey’s correction. Data are presented as mean ± standard deviation (SD), *p < 0.05, **p < 0.01, ***p < 0.001, n.s. non-significant.

To further determine the role of NLRP3 in the development of trained immunity following CMI, we used microglial conditional NLRP3 knockout mice (CX3CR1^CreER^ × NLRP3^fl/fl^ mice). The depletion of microglial NLRP3 was induced by tamoxifen administration after microinfarction (Fig. 5A, B and Fig. S6D–F). Interestingly, the elevated H3K4me1 and H3K4me3 expression levels by CMI were abolished in ${{NLRP}3}_{{cxc}3r1}^{-/-}$ mice (Fig. 5C and Fig. S6G, H). And microglial NLRP3 knockout abolished H3K4me3 modification on TNF-α and IL-1β promoters (Fig. 5D–G).

Most importantly, CMI-increased infarct size was mitigated in ${{NLRP}3}_{{cxc}3r1}^{-/-}$ mice seven days post PT stroke (Fig. 5H, I and Fig. S6I, J), while the microglia also showed mitigated activation, indicating by the reduced number and filament length. (Fig. 5J–L).

### Propagation of inflammatory cytokines after CMI initiates trained immunity in photothrombotic stroke cortex

**Fig. 6:**
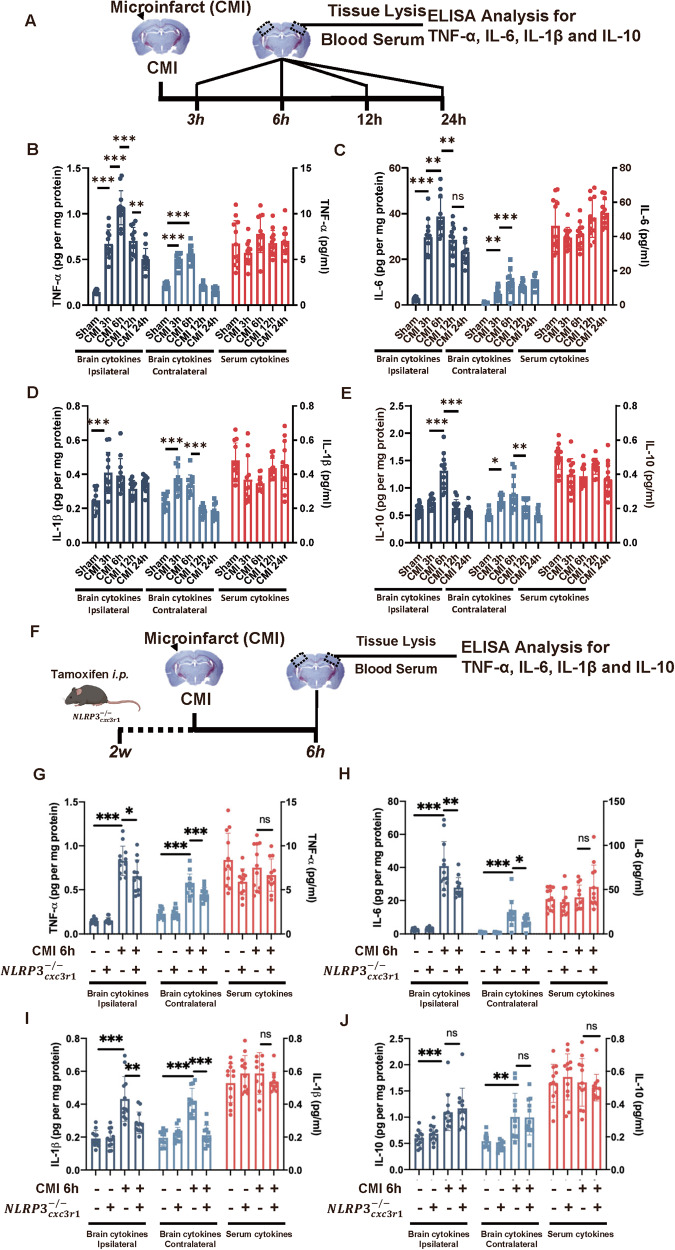
Propagation of inflammatory cytokines after CMI initiates trained immunity in photothrombotic stroke cortex. **A** Schematic diagram of experimental design. Quantitative ELISA analysis of TNF-α (**B**), IL-6 (**C**), IL-1β (**D**), and IL-10 (**E**) expression in ipsilateral cortex, contralateral cortex, and peripheral serum after PT stroke, *n* = 12 mice per group. One-way ANOVA was used to analyze the data. For TNF-α analysis, ipsilateral cytokines: *F*_4, 55_ = 70.40, *p* < 0.0001; Sham vs. CMI 6h: *p* < 0.0001; Contralateral cytokines: *F*_4, 55_ = 147.1, *p* < 0.0001; Sham vs. CMI 6h: *p* < 0.0001. Serum cytokines: *F*_4, 55_ = 2.047, *p* = 0.1004; Sham vs. CMI 3h: *p* = 0.6059 (**B**). For IL-6 analysis, ipsilateral cytokines: *F*_4, 55_ = 56.01, *p* < 0.0001; Sham vs. CMI 6h: *p* < 0.0001. Contralateral cytokines: *F*_4, 55_ = 23.23, *p* < 0.0001; Sham vs. CMI 6h: *p* < 0.0001. Serum cytokines: *F*_4, 55_ = 4.546, *p* = 0.0030; Sham vs. CMI 6h: *p* = 0.7525 (**C**). For IL-1βanalysis, ipsilateral cytokines: *F*_4, 55_ = 7.952, *p* < 0.0001; Sham vs. CMI 6h: *p* = 0.0004. Contralateral cytokines: *F*_4, 55_ = 34.73, *p* < 0.0001; Sham vs. CMI 6h: *p* < 0.0001. Serum cytokines: *F*_4, 55_ = 3.635, *p* = 0.0107; Sham vs. CMI 6h: *p* = 0.0207 (**D**). For IL-10 analysis, ipsilateral cytokines: *F*_4, 55_ = 35.99, *p* < 0.001; Sham vs. CMI 6h: *p* < 0.0001. Contralateral cytokines: *F*_4, 55_ = 11.86, *p* < 0.0001; Sham vs. CMI 6h: *p* < 0.0001. Serum cytokines: *F*_4, 55_ = 5.919, *p* = 0.0005; Sham vs. CMI 6h: *p* = 0.0047 (**E**) with Tukey’s correction. **F** Schematic diagram of experimental design. Quantitative ELISA analysis of TNF-α (**G**), IL-6 (**H**), IL-1β (**I**), and IL-10 (**J**) expression in ipsilateral cortex, contralateral cortex, and peripheral serum after PT stroke in WT and ${{NLRP}3}_{{cxc}3r1}^{-/-}$ mice. *n* = 12 mice per group. Two-way ANOVA for analysis. Ipsilateral cytokines: for interaction: *p* = 0.0166. So t-tests was used to detect the difference between groups. CMI vs CMI+${{NLRP}3}_{{cxc}3r1}^{-/-}$: *p* = 0.0233. Contralateral cytokines: for interaction: *p* = 0.0489. CMI vs. CMI+${{NLRP}3}_{{cxc}3r1}^{-/-}$: *p* < 0.0001. Serum cytokine: for interaction: *p* = 0.2285. CMI vs. CMI+${{NLRP}3}_{{cxc}3r1}^{-/-}$: *p* = 0.8071 (**G**); For IL-6 analysis, ipsilateral cytokines: for interaction: *p* = 0.0052. CMI vs. CMI+${{NLRP}3}_{{cxc}3r1}^{-/-}$: *p* = 0.0100. Contralateral cytokines: for interaction: *p* = 0.0544. CMI vs. CMI+${{NLRP}3}_{{cxc}3r1}^{-/-}$: *p* = 0.0317. Serum cytokine: for interaction: *p* = 0.1273. CMI vs. CMI+${{NLRP}3}_{{cxc}3r1}^{-/-}$: *p* = 0.3334 (**H**); For IL-1βanalysis, ipsilateral cytokines: for interaction: *p* = 0.0040. CMI vs. CMI+${{NLRP}3}_{{cxc}3r1}^{-/-}$: *p* = 0.0031. Contralateral cytokines: for interaction: *p* < 0.0001. CMI vs. CMI+${{NLRP}3}_{{cxc}3r1}^{-/-}$: *p* < 0.0001. Serum cytokine: for interaction: *p* = 0.0774. CMI vs. CMI+${{NLRP}3}_{{cxc}3r1}^{-/-}$: *p* = 0.6185 (**I**); For IL-10 analysis, ipsilateral cytokines: for interaction: *p* = 0.9477. CMI vs. CMI+${{NLRP}3}_{{cxc}3r1}^{-/-}$: *p* = 0.9222. Contralateral cytokines: for interaction: *p* = 0.6117. CMI vs. CMI+${{NLRP}3}_{{cxc}3r1}^{-/-}$: *p* = 0.9997. Serum cytokine: for interaction: *p* = 0.3239. CMI vs. CMI+${{NLRP}3}_{{cxc}3r1}^{-/-}$: *p* = 0.9192 (**J**) with Turkey’s corrections. Data are presented as mean ± standard deviation (SD), *p < 0.05, **p < 0.01, ***p < 0.001, n.s. non-significant.

Although we verified that CMI could potentiate the detrimental effect of recurrent stroke through trained immunity in NLRP3-dependent manner, it is still unclear how CMI influenced the PT stroke in the contralateral side. Classically, trained immunity is induced by vaccinations and inflammatory stimulators, such as LPS [21]. Therefore, pro-inflammatory cytokines, such as TNF-α and IL-1β, in the acute stage of CMI might mediate the activation of trained immunity. The inflammatory responses after CMI were determined. The inflammatory cytokine TNF-α, IL-6, IL-1β, and IL-10 levels were elevated and peaked 6 h after microinfarct in both ipsilateral and contralateral cortexes, whereas serum cytokines did not show significant changes (Fig. 6A–E). Interestingly, milder inflammatory responses were observed on the contralateral side at the same time (Fig. 6D–E), indicating that CMI was able to propagate whole-cortex inflammatory responses and these inflammatory cytokines might be the trigger to initiate the trained immunity in the contralateral cortex.

Using ${{NLRP}3}_{{cxc}3r1}^{-/-}$ mice, we further investigated the cellular sources of these inflammatory cytokines. The microglial knockout of NLRP3 significantly abolished the elevation of IL-1β in both ipsilateral and contralateral cortexes (Fig. 6F–J). Similarly, there was no alteration in serum cytokines observed, either with CMI or in the case of microglia NLRP3 knockout. As we previously found that microglial knockout of NLRP3 could abolish the trained effect on the contralateral cortex (Fig. 5C–H), it was reasonable to speculate that the activation of trained immunity on the contralateral cortex after CMIs was primed by the microglial inflammation from the ipsilateral cortex which was at least induced by the release of IL-1β.

## Discussion

The present study found that CMI increases the neuropathology of recurrent ischemic stroke via NLRP3-dependent trained immunity. CMI induced MLL1-dependent H3K4 methylation, increased the inflammatory responses, and exacerbated the outcome of subsequent PT stroke. The interaction between nuclear NLRP3 and MLL1 is critical in CMI-induced trained immunity as the knockout of microglial NLRP3 inhibits trained immunity and attenuates recurrent stroke. The study results also highlighted the influence of trained immunity on recurrent stroke and provided a novel insight on the influence of NLRP3 in this innate immunity memory formation.

It is common for elderly individuals to experience CMIs [22]. However, recurrent stroke risk is difficult to predict, as most CMIs cannot be detected via magnetic resonance imaging. It has been recently reported that diffusion-weighted imaging can detect and monitor acute CMIs for 2 weeks, which may provide a better intervention window [23]. The present study found that a single CMI can induce epigenetic alterations (H3K4me1 and H3K4me3) in the contralateral cortex, suggesting that a single CMI may have a substantial impact on the brain at the epigenetic level. This impact is significant as it induces trained immunity, which leads to excessive immune activation and aggravates the consequences of subsequent strokes.

It is not clear how CMIs induce trained immunity. Traditionally, it is triggered by vaccinations and inflammatory stimulators, such as LPS [21]. However, endogenous alarm signals associated with tissue damage and sterile inflammation can also induce trained immunity through the epigenetic regulation of histone modifications, such as inflammatory cytokines, damage‐associated molecular patterns (DAMPs), Oxidized Low-density Lipoprotein (oxLDL), and High-density Lipoprotein HDL [24]. It has been reported that DAMPs activate TLR2 to epigenetically modify histone methylation, while peripheral low-dose TNF-α can activate trained immunity in the brain [25]. Consistently, the present study results demonstrated that TNF-α, IL-6, and IL-1β levels were elevated in the contralateral cortex in a similar but milder pattern to that in the ipsilateral CMI cortex, which indicated that CMI-induced trained immunity was based on the release of inflammatory cytokines. Alternatively, released inflammatory cytokines from CMIs might reach the contralateral cortex through cerebrospinal fluid, as evidenced by the fact that peripheral serum inflammatory cytokine levels did not elevate after CMI. The epigenetic modification caused by this damage signal further worsens the outcome of subsequent strokes.

Ischemic preconditioning (IPC) demonstrates an impressive capacity to protect against future strokes [26]. IPC could be induced not only by transient cerebral ischemic event (TIA) but also by ischemia. In addition to trigger an elevation in HIF-1a expression in both neurons and astrocytes, IPC is also reported to reduce H3K4 methylation [27, 28]. In contrast, CMI can increase the H3K4 methylation and promote the epigenetic modification in proinflammatory-related gene, indicating completely different signaling pathways underlying these two conditions [29].

Histone modifications with chromatin reconfiguration are a central process in trained immunity [30]. As a super-enhancer, H3K4me1/3 modifications unfold the chromatin, enabling easier transcription of the modified gene sequences. This deposition of H3K4 methylation is mainly accomplished by MLL family histone methyltransferases, particularly MLL1 [31]. In the present study, CMI induced the interaction between MLL1 and NLRP3 in microglial nuclei. Interestingly, the interaction did not lead to the activation of the NLRP3 inflammasome. The IL-1β production remained unchanged in microglia after a single CMI. Instead, the interaction was related to an increase in H3K4me1 and H3K4me3 expression. Furthermore, a knockout of microglial NLRP3 significantly inhibited MLL1 elevation, suggesting that NLRP3 mediated CMI-induced trained immunity through the interaction with MLL1 independently of inflammasomes. Indeed, NLRP3 has been reported to have several inflammasome-independent functions in immunity, although IL-1β and IL-18 secretion from activated NLRP3 inflammasomes is important for various innate and adaptive immune responses. For example, NLRP3 can interact with both DNA and transcription factor IRF4 to mediate the differentiation of Th2 cells [32].

NLRP3 has been a therapeutic target in the treatment of cerebrovascular diseases [33]. However, it is debated whether NLRP3 inhibition can reduce infarct volume in experimental ischemic stroke [34]. Previously, our group found that NLRP3 could aggravate the recurrent ischemic strokes following a single infarction in an inflammasome-dependent manner [35]. The data from this study further revealed that NLRP3 had an inflammasome-independent role in exacerbating subsequent stroke. Therefore, NLPR3 is a critical candidate to influence the recurrent ischemic strokes and proper therapeutic intervention is needed to rectify this abnormal immune activation.

There are some limitations in the present study. Although we found that IL-1β could induce the trained immunity in the contralateral cortex after CMI, whether the release of other inflammatory cytokines, such as IL-6, TNF-α, could promote the formation of trained immunity worth further study. Besides, further research is required to address the function of caspase-1 and ASC in microglial nuclei. Researchers should also investigate other functional roles of NLRP3 in the nuclei of inflammatory cells. Moreover, microinfarcts are just one type of vascular disease and the influence of other small vascular diseases (SVDs) on recurrent stroke requires more research.

The present study is the first to reveal the epigenetic regulation role of NLRP3 in innate immune memory, as it increases H3K4me1 and H3K4me3 levels through its interaction with the MLL1 complex. This innate immune memory formation influences the pathology of recurrent stroke in mice with CMI. Therefore, NLRP3 may provide an alternative therapeutic target to mitigate the detrimental effect of CMI and subsequent stroke.

## Conclusions

The present study confirmed that a single CMI event can significantly exacerbate the outcome of subsequent stroke. This relationship was likely present because the microinfarct triggered the formation of innate immunity memory in microglia in a NLRP3-dependent manner, which epigenetically primes the inflammatory genes ready for transcription after the microinfarct. Our results highlighted the clinical relevance of microinfarct on subsequent stroke and pointed out the importance of NLRP3 as a potential therapeutic target to mitigate recurrent stroke in CMI patients.

### Supplementary information

Supplementary information

aj-checklist

Suplemantery material---WB

Original Data File

## Data Availability

The datasets generated and/or analyzed during the current study are available in the supplementary material.
