# Supplementary material for: Cortical microinfarcts potentiate recurrent ischemic injury through NLRP3-dependent trained immunity
Source: Cell Death Dis. 2024 Jan 12;15(1):36. doi: 10.1038/s41419-023-06414-7 (PMC10786939; doi:10.1038/s41419-023-06414-7)
Supplement: Supplementary file 1 — Supplementary information [file 41419_2023_6414_MOESM1_ESM.docx]

*Supplementary information*

**Cortical Microinfarcts Potentiate Recurrent Ischemic Injury through NLRP3-Dependent Trained Immunity**

Yiwei Feng et al.


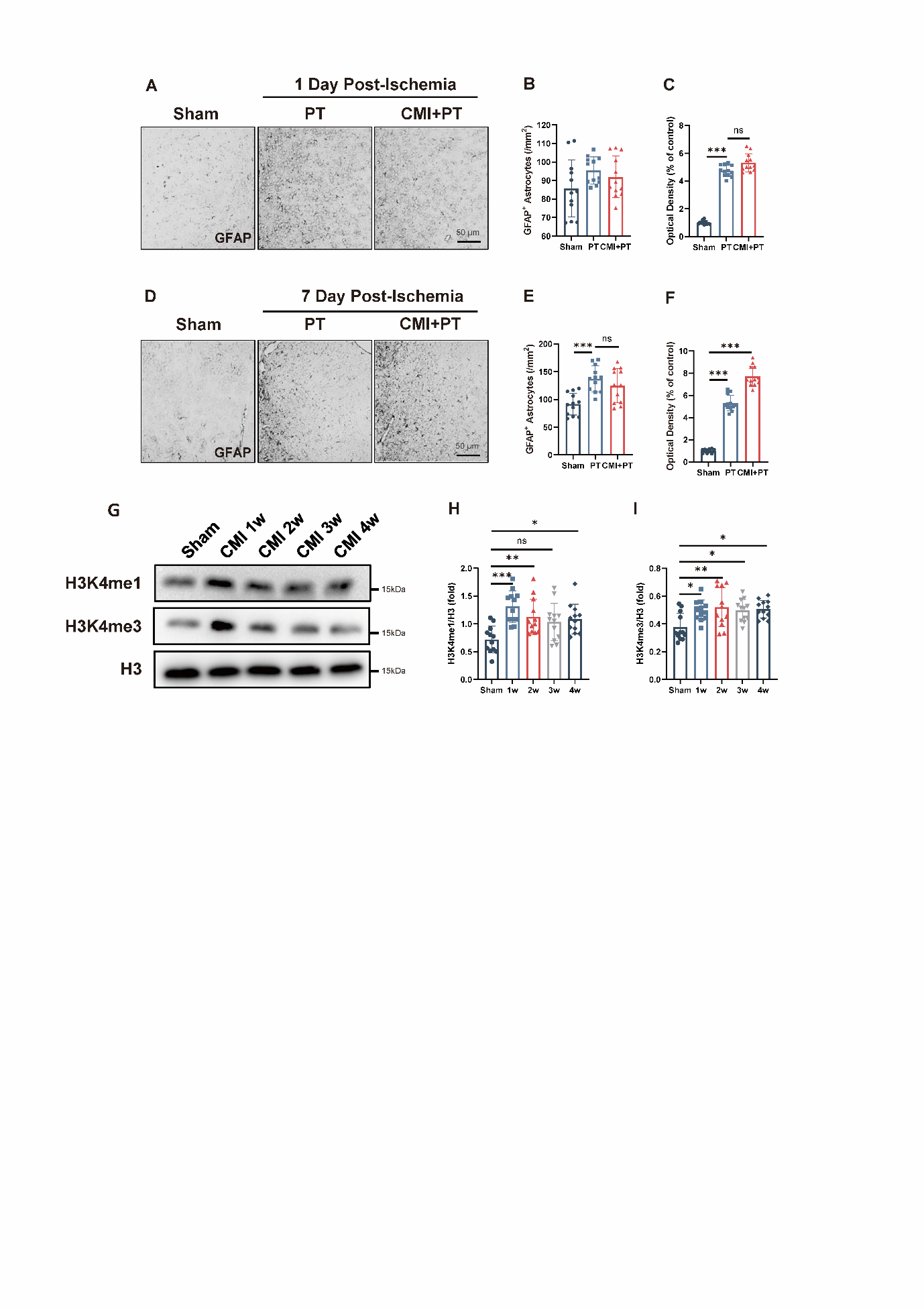


**Figure S1.** **A-C** Representative GFAP IHC staining (**A**) and quantitative analysis (**B, C**) of GFAP-positive astrocytes around para-infarct area one day post PT stroke. One-way ANOVA *F*_2, 33_ = 2.117, *p* = 0.1365 (**B**) with Tukey’s correction. One-way ANOVA *F*_2, 33_ = 331.2, *p* < 0.001 (**C**) with Tukey’s correction. Scale bars, 50 μm. **D-F**  Representative GFAP IHC staining (**D**) and quantitative analysis (**E, F**) of GFAP-positive astrocytes around para-infarct area seven days post PT stroke. One-way ANOVA. *F*_2, 33_ = 10.7, *p* = 0.0003. Sham vs PT: *p* = 0.0003 (**E**) with Tukey’s correction. One-way ANOVA. *F*_2, 33_ = 310.4, *p* < 0.001 (**F**) with Tukey’s correction. Scale bars, 50 μm. **G–I** Representative immunoblot (G) and quantitative analysis of H3K4me1 (**H**) and H3K4me3 expression (**I**), n = 12 mice per group. One-way ANOVA *F*_4, 55_ = 6.527, *p =* 0.0002. Sham vs CMI 1w: *p* < 0.0001; Sham vs CMI 2w: *p* = 0.0096; Sham vs CMI 3w: *p* = 0.0768; Sham vs CMI 4w: *p* = 0.0247 (**H**) and *F*_4, 55_ = 4.491, *p* = 0.0033; Sham vs CMI 1w: *p* = 0.0254; Sham vs CMI 2w: *p* = 0.0045; Sham vs CMI 3w: *p* = 0.0233; Sham vs CMI 4w: *p* = 0.0128 (**I**) with Tukey’s correction. Data are presented as mean ± standard deviation (SD), **p* < 0.05, ***p* < 0.01, ****p* < 0.001, ns non-significant.

**
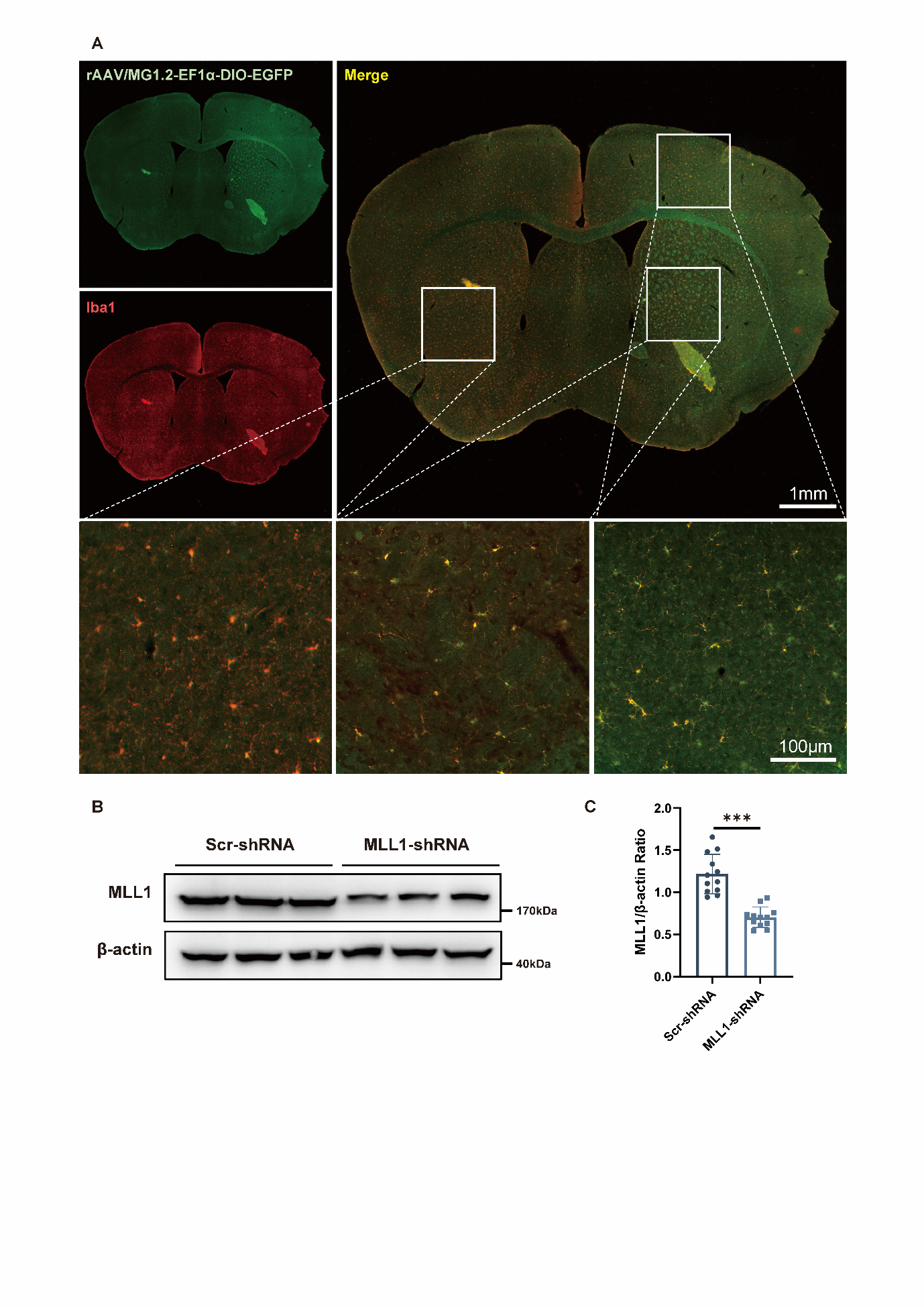
**

**Figure S2.** **A** Representative image of rAAV/MG1.2-EF1α-DIO-EGFP injection. The EGFP (green) is co-stained with Iba-1(red), indicating the rAAV is expressed in the microglia cells. **B, C** Representative immunoblot of MLL1 expression (**B**) and quantification of MLL1 expression (**C**). T-test, *p* < 0.0001. Data are presented as mean ± standard deviation (SD), **p* < 0.05, ***p* < 0.01, ****p* < 0.001, ns non-significant.


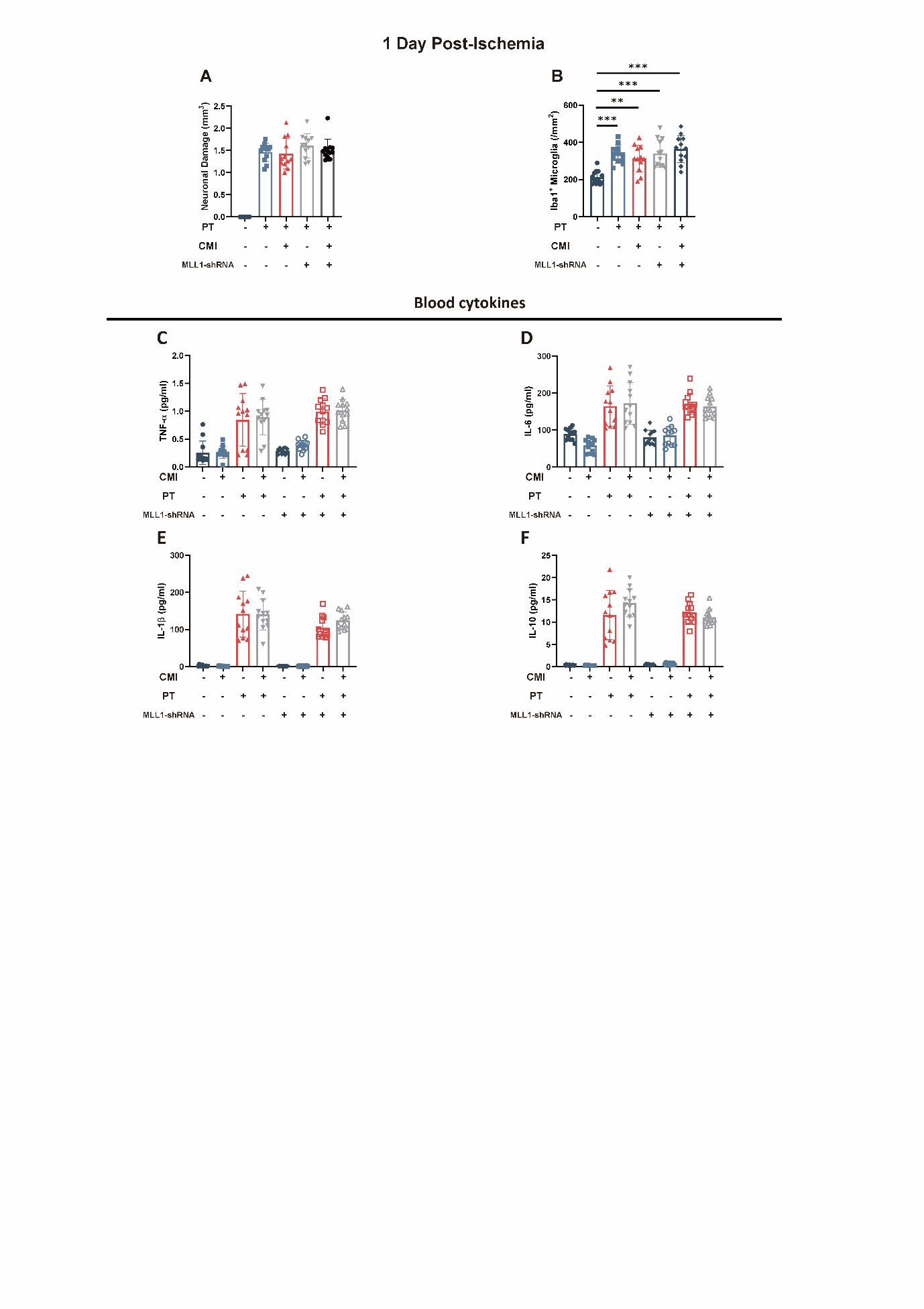


**Figure S3**. **A, B** Quantitative analysis of Nissl (**A**) and Iba1 IHC (**B**) staining showing that MLL1-shRNA treatment did not influence infarct size and microglial activation one day post PT stroke. Two-way ANOVA. For interactions: *p* = 0.7052. PT+CMI vs PT+CMI+MLL1-shRNA: *p* = 0.8797 (**A**) For interactions: *p* = 0.2334. PT+CMI vs PT+CMI+ MLL1-shRNA: *p* = 0.2640 (**B**) with Tukey’s correction. **C–F** Quantitative analysis of ELISA results showed that TNF-α (**C**), IL-6 (**D**), IL-1β (**E**), and IL-10 (**F**) expression in peripheral blood did not deteriorate in stroke mice with preceding microinfarct, (n = 12 mice per group). Three-way ANOVA, for interactions: *p* = 0.5518. PT vs CMI+PT: *p* = 0.9997 (**C**); for interactions: *p* = 0.0742. PT vs CMI+PT: *p* = 0.9996 (**D**); for interactions: *p* = 0.4190. PT vs CMI+PT: *p* > 0.9999 (**E**); and for interactions: *p* = 0.0511. PT vs CMI+PT: *p* = 0.1473 (**F**) with Tukey’s correction. Data are presented as mean ± standard deviation (SD), **p* < 0.05, ***p* < 0.01, ****p* < 0.001, ns non-significant.

**
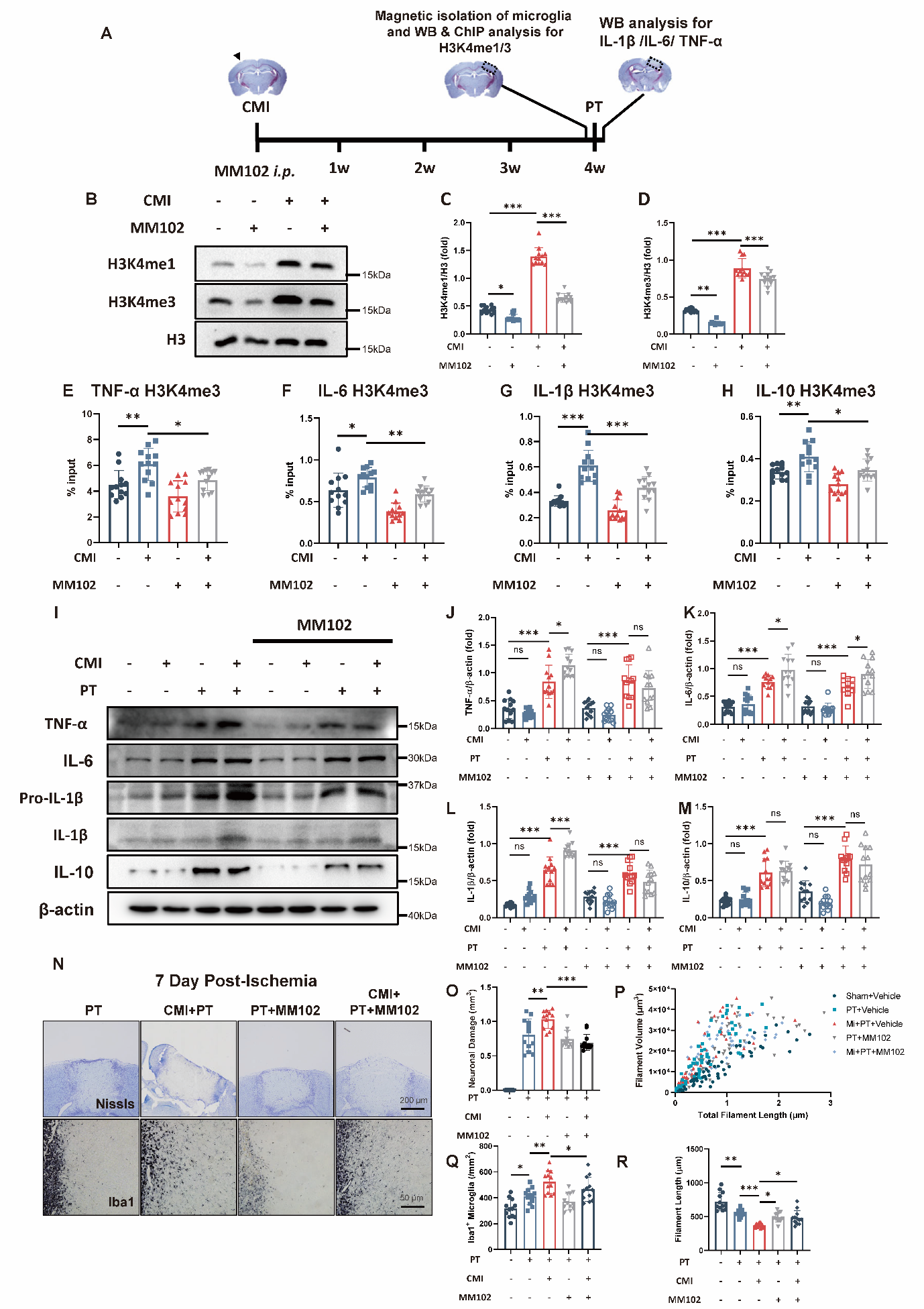
**

**Figure S4.** **A** Schematic diagram of experimental design. **B–D** Immunoblots and quantitative analysis of H3K4me1/3 showing microglial H3K4me1/3 expression in contralateral mouse cortex subjected to CMI and MM102 treatment, n = 12 mice per group. Two-way ANOVA, for interaction: *p* < 0.0001. So t-tests was used to detect the difference between groups. Sham vs CMI: *p* < 0.0001; CMI vs CMI+MM102: *p* < 0.0001 (**C**) For interaction: *p* = 0.592. For MM102 factor: *p* < 0. 0001.For CMI factor: *p* < 0.0001. Sham vs CMI: *p* < 0.0001; CMI vs CMI+MM102: *p* = 0.0004 (**D**) with Turkey’s correction. **E–H** Quantitative analysis of H3K4me3 ChIP-qPCR on TNF-α (**E**), IL-6 (**F**), IL-1β (**G**), and IL-10 (**H**) promoters in contralateral microglia 4 weeks after CMI and MM102 treatment, n = 12 mice per group. Two-way ANOVA, for interaction *p* = 0.5157. For MM102 factor, *p* = 0.0017. CMI vs CMI+MM102: *p* = 0.0458 (**E**) For interaction *p* = 0.551. For MM102 factor, *p* < 0.0001. CMI vs CMI+MM102: *p* = 0.0042 (**F**); For interaction *p* = 0.6271. For MM102 factor, *p* < 0.0001. CMI vs CMI+MM102: *p* = 0.0001 (**G**); For interaction *p* = 0.8529. For MM102 factor, *p* < 0.0959. CMI vs CMI+MM102: *p* = 0.7085 (**H**) with Tukey’s correction. **I–M** Immunoblots and quantitative analysis of TNF-α (**J**), IL-6 (**K**), IL-1β (**L**), and IL-10 (**M**) showing pro-inflammatory cytokine expression in peri-infarct region 12 h after PT stroke, n = 12 mice per group. Three-way ANOVA. For interaction, *p* = 0.0311. So t-tests was used to detect the difference between groups. PT vs CMI+PT: *p* = 0.0103; CMI+PT vs CMI+PT+MM102: *p* = 0.0011 (**J**); For interaction, *p* = 0.4667. PT vs CMI+PT: *p* = 0.0499; CMI+PT vs CMI+PT+MM102: *p* = 0.9571 (**K**); For interaction, *p* = 0.0349. So t-tests was used to detect the difference between groups. PT vs CMI+PT: *p* = 0.0001; CMI+PT vs CMI+PT+MM102: *p* < 0.0001 (**L)** For interaction, *p* = 0.5228. PT vs CMI+PT: *p* > 0.9999; CMI+PT vs CMI+PT+MM102: *p* = 0.8485 (**M**) with Tukey’s correction. **N–R** Representative Nissl and Iba1 IHC staining (**N**) and quantitative analysis of infarct size and microglial activation (**O–R**) showing PT stroke exacerbated by CMI was potentially mitigated by H3 methylation inhibition, n = 12 mice per group. Two-way ANOVA. For interaction, *p* = 0.0045. So t-tests was used to detect the difference between groups. CMI+PT vs CMI+PT+MM102: *p* < 0.0001 (**O**); For interaction, *p* = 0.7189. CMI+PT vs CMI+PT+MM102: *p* = 0.2927 (**Q**); For interaction, *p* = 0.0001. So t-tests was used to detect the difference between groups. CMI+PT vs CMI+PT+MM102: *p* = 0.0017 (**R**) with Tukey’s correction. Scale bar, 200 μm for Nissl staining and 50 μm for Iba1 staining. Data are presented as mean ± standard deviation (SD), **p* < 0.05, ***p* < 0.01, ****p* < 0.001, n.s., non-significant.


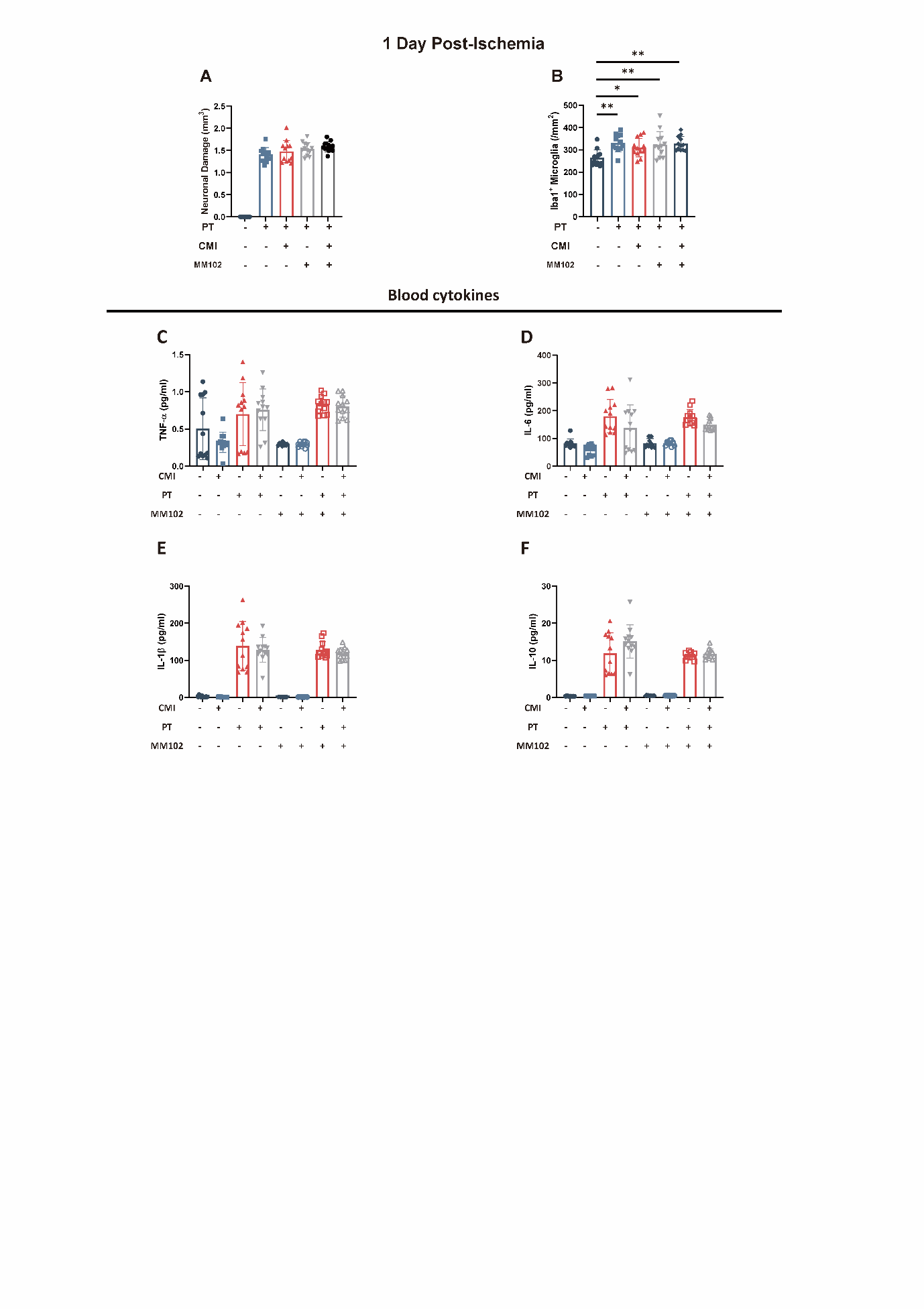
**Figure S5. A, B** Quantitative analysis of Nissl (**A**) and Iba1 IHC (**B**) staining showing that H3 methylation did not influence infarct size and microglial activation one day post PT stroke. Two-way ANOVA. For interactions: *p* = 0.8229. PT+CMI vs PT+CMI+MM102: *p* = 0.4620 (**A**) and Two-way ANOVA. For interactions: *p* = 0.3339. PT+CMI vs PT+CMI+MM102: *p* = 0.7648 (**B**) with Tukey’s correction. **C–F** Quantitative analysis of ELISA results showed that TNF-α (**C**), IL-6 (**D**), IL-1β (**E**), and IL-10 (**F**) expression in peripheral blood did not deteriorate in stroke mice with preceding microinfarct, (n = 12 mice per group). Three-way ANOVA, for interactions: *p* = 0.1884. PT vs CMI+PT: *p* = 0.9988 (**C**); for interactions: *p* = 0.8568. PT vs CMI+PT: *p* = 0.1667 (**D**); for interactions: *p* = 0.9968. PT vs CMI+PT: *p* = 0.9773 (**E**); and for interactions: *p* = 0.2261. PT vs CMI+PT: *p* = 0.0602 (**F**) with Tukey’s correction. Data are presented as mean ± standard deviation (SD), **p* < 0.05, ***p* < 0.01, ****p* < 0.001, ns non-significant.


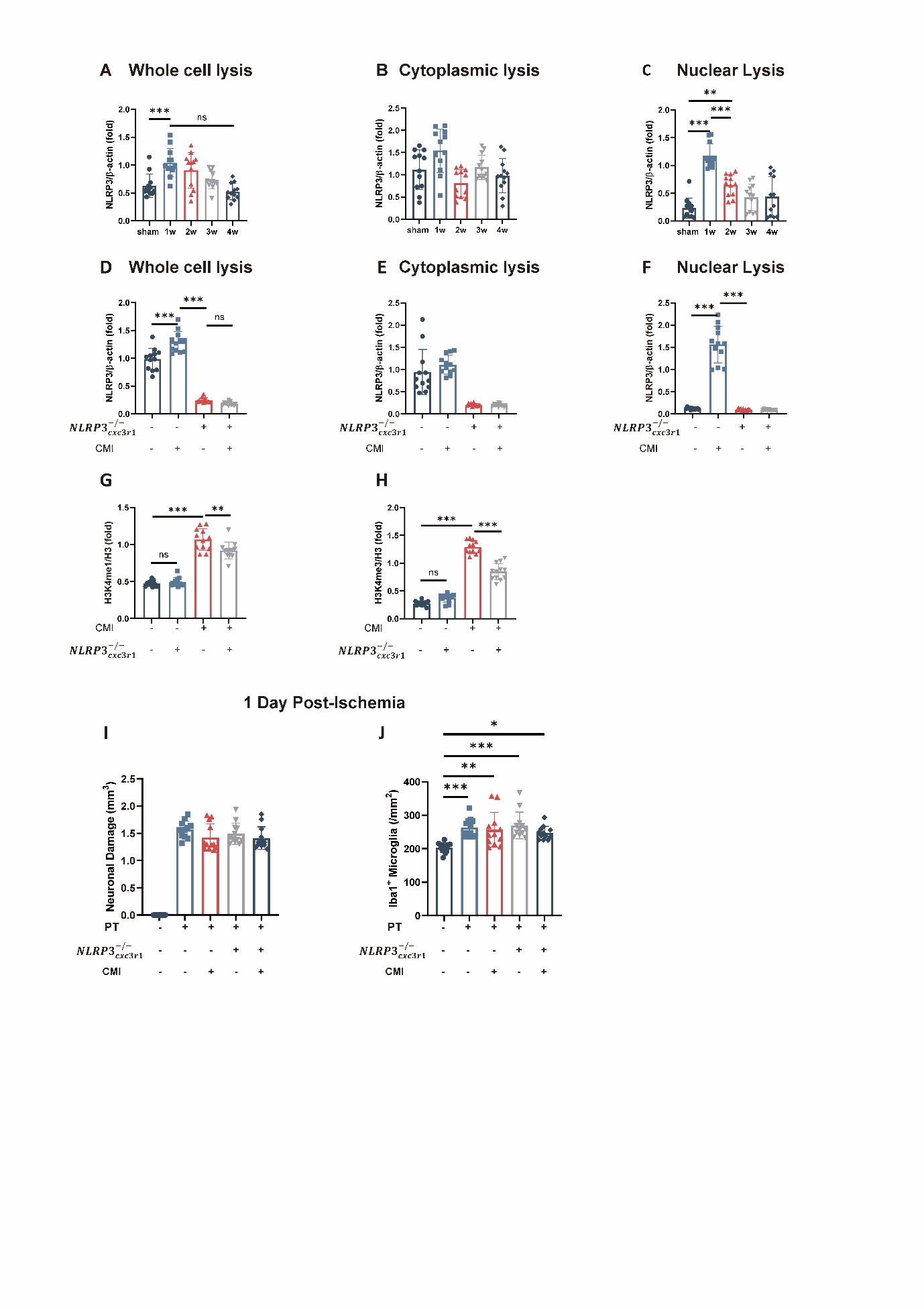


**Figure S6. A–C** Quantitative analysis of NLRP3 expression in whole-cell lysis (**A**), cytoplasmic lysis (**B**), and nuclear lysis (**C**) following CMI, n = 12 mice per group. One-way ANOVA *F*_4, 55_ = 10.17, *p* < 0.0001; Sham vs 1w: *p* = 0.0004 (**A**); *F*_4, 55_ = 5.86, *p* = 0.0005; Sham vs 1w: *p* = 0.0691 (**B**); and *F*_4, 55_ = 25.88, *p* < 0.0001; Sham vs 1w: *p* < 0.0001 (**C**) with Tukey’s correction. **D–F** Quantitative analysis of NLRP3 expression in whole-cell lysis (**D**), cytoplasmic lysis (**E**), and nuclear lysis (**F**) in WT and ${NLRP3}_{cxc3r1}^{-/-}$ mice, n = 12 mice per group. Two-way ANOVA, for interaction: *p* < 0.0001; CMI vs CMI+${NLRP3}_{cxc3r1}^{-/-}$ : *p* < 0.0001 (**D**); for interaction: *p* = 0.2826; CMI vs CMI+${NLRP3}_{cxc3r1}^{-/-}$ : *p* < 0.0001 (**E**); for interaction: *p* < 0.0001;CMI vs CMI+${NLRP3}_{cxc3r1}^{-/-}$ : *p* < 0.0001 (**F**) with Tukey’s correction. **G, H** Quantitative analysis of H3K4me1 and H3K4me3 expression showing that microglial NLRP3 knockout significantly inhibited H3 methylation, n = 12 mice per group. Two-way ANOVA, for interaction: *p* = 0.0071; CMI vs CMI+${NLRP3}_{cxc3r1}^{-/-}$ :*p* = 0.0043 (**G**) and for interaction: *p* < 0.0001; CMI vs CMI+${NLRP3}_{cxc3r1}^{-/-}$ : *p* < 0.0001(**H**) with Tukey’s correction. **I, J** Quantitative analysis of Nissl and Iba1 IHC staining showing that microglial NLRP3 knockout did not influence infarct size and microglial activation one day post PT stroke, n = 12 mice per group. Two-way ANOVA, for interaction: *p* = 0.5658; CMI vs CMI+${NLRP3}_{cxc3r1}^{-/-}$ : *p* = 0.9992 (**I**) and for interaction: *p* = 0.4707; CMI vs CMI+${NLRP3}_{cxc3r1}^{-/-}$ : *p* = 0.9281 (**J**) with Tukey’s correction. Data are presented as mean ± standard deviation (SD), **p* < 0.05, ***p* < 0.01, ****p* < 0.001, ns non-significant.


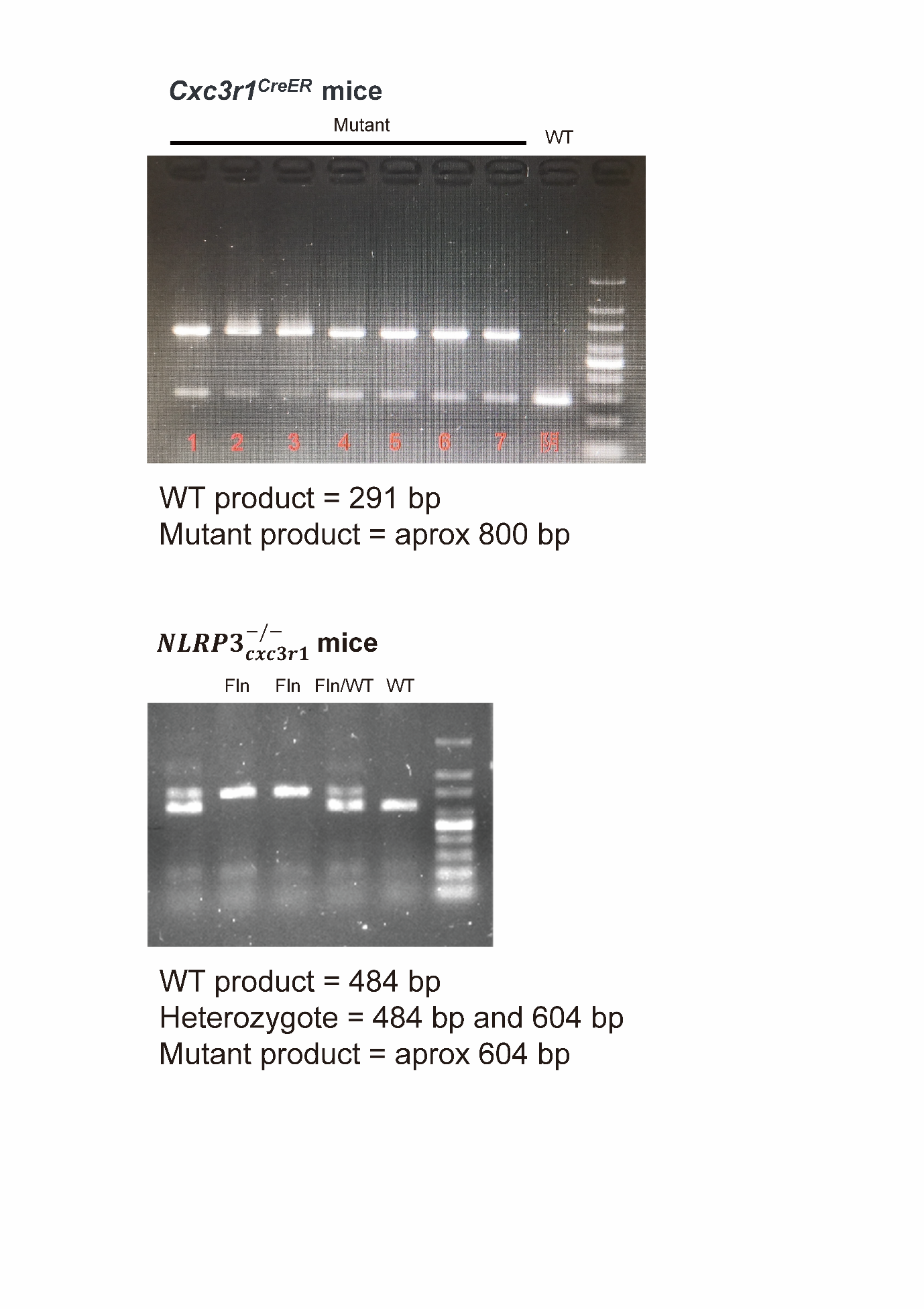


**Figure S7.** Genotyping for Cx3cr1CreER and ${NLRP3}_{cxc3r1}^{-/-}$ mice.
