# Supplementary material for: Cortical microinfarcts potentiate recurrent ischemic injury through NLRP3-dependent trained immunity
Source: Cell Death Dis. 2024 Jan 12;15(1):36. doi: 10.1038/s41419-023-06414-7 (PMC10786939; doi:10.1038/s41419-023-06414-7)

Full and uncropped western blot for Figure 3B

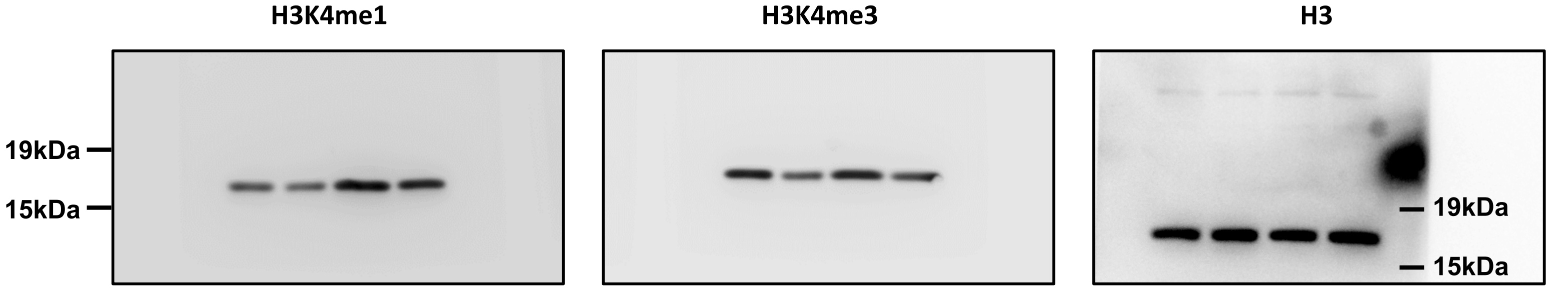

Full and uncropped western blot for Figure 3I

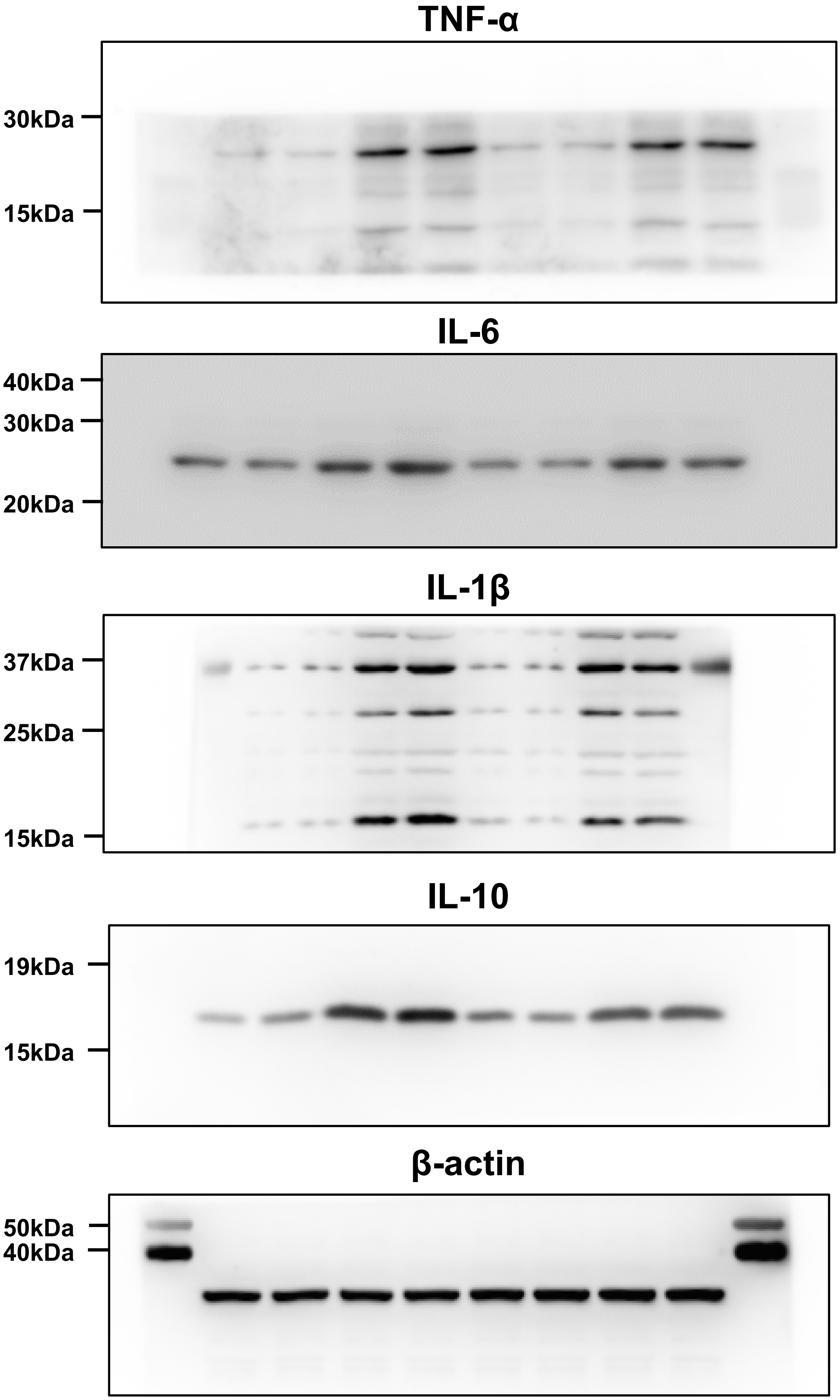

Full and uncropped western blot for Figure 4A

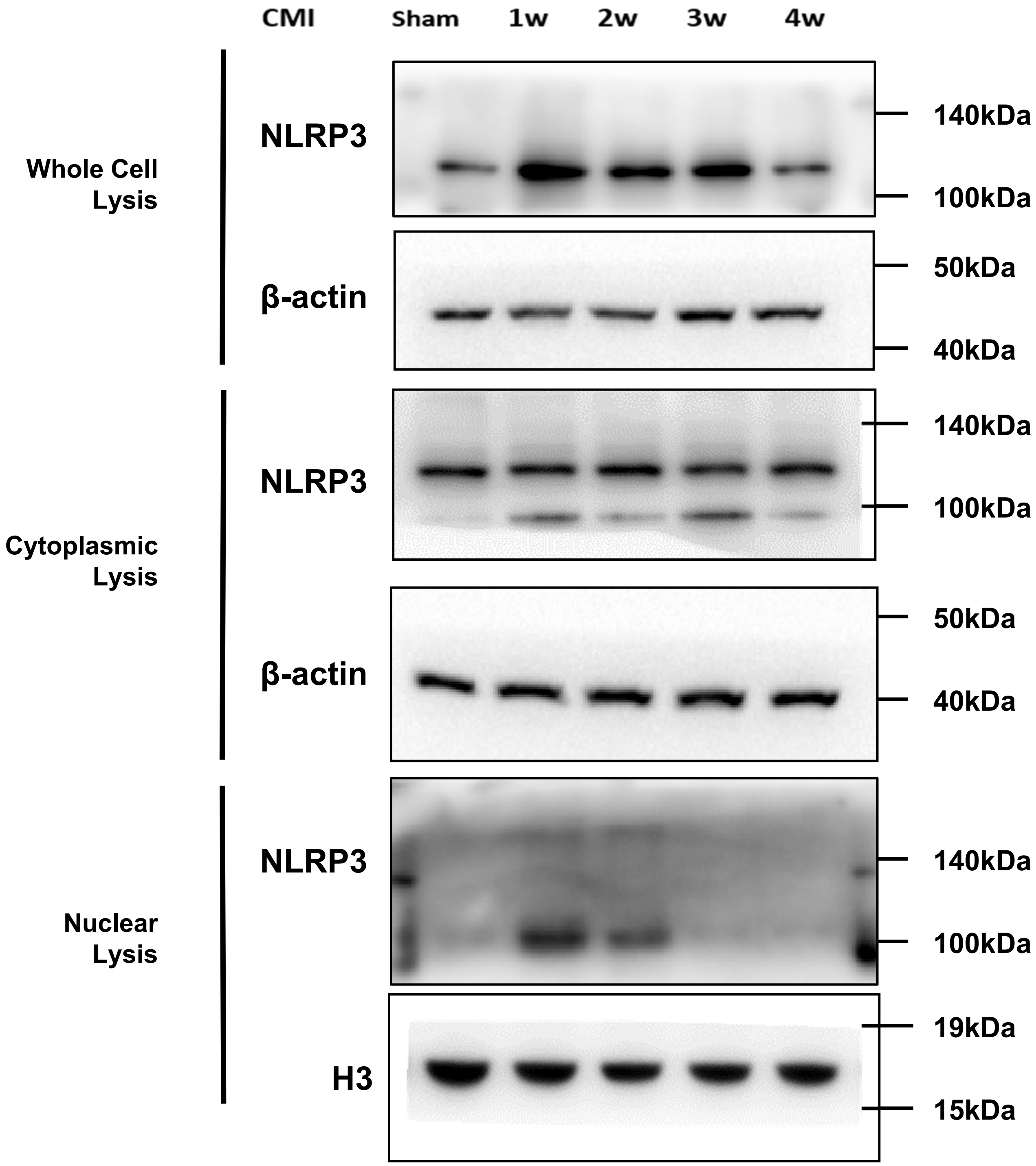

Full and uncropped western blot for Figure 4G

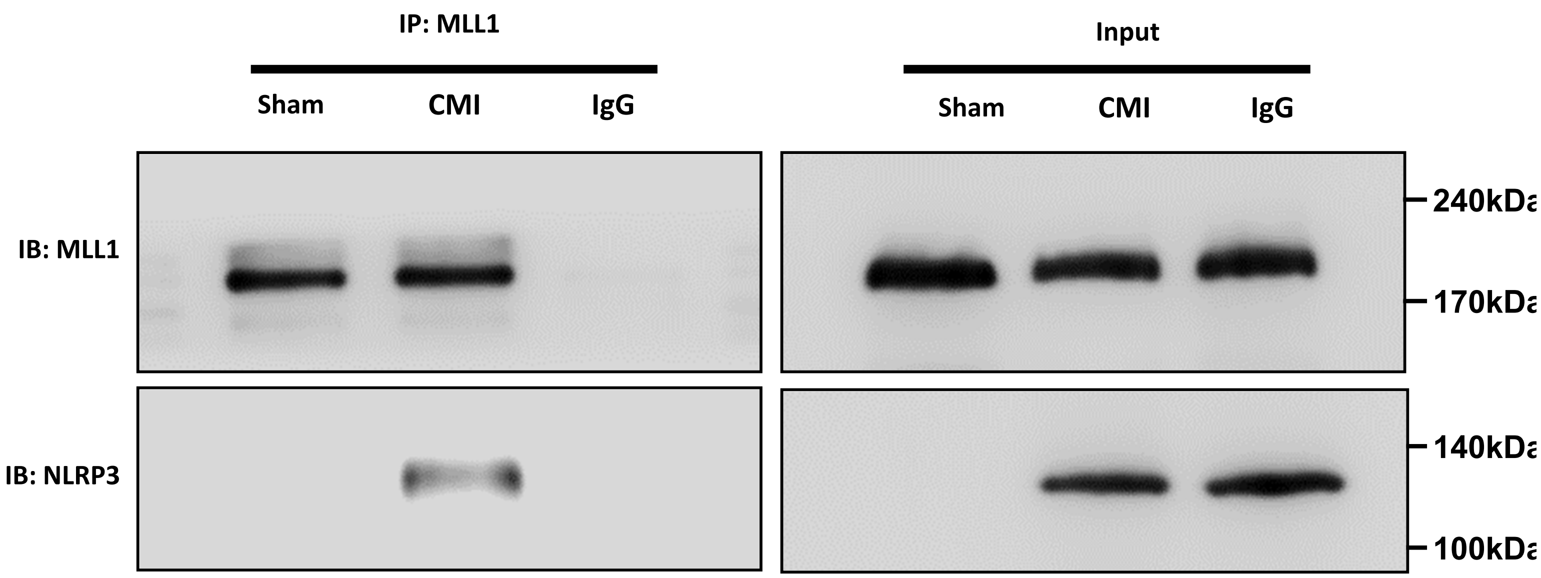

Full and uncropped western blot for Figure 4K

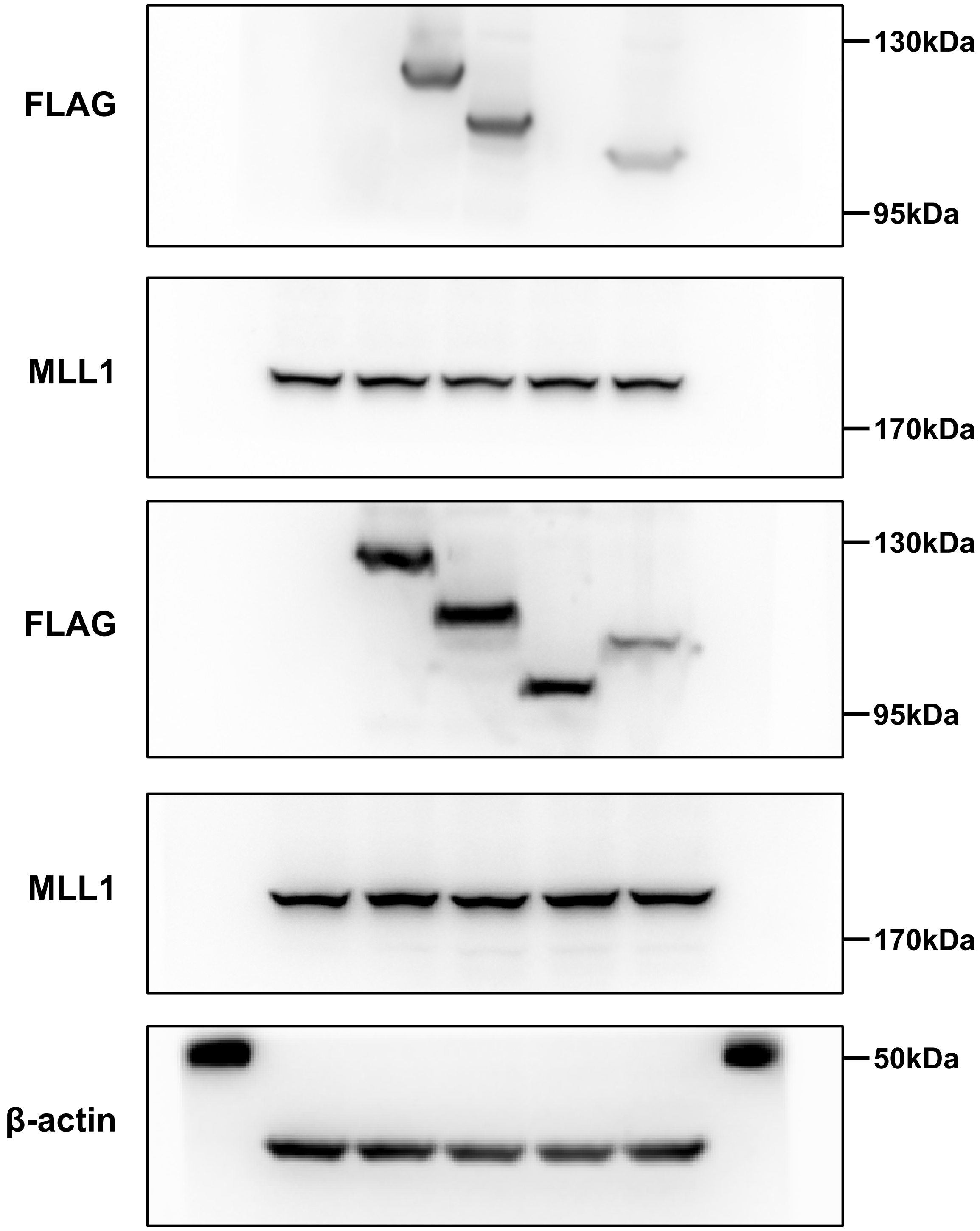

Full and uncropped western blot for Figure 5B

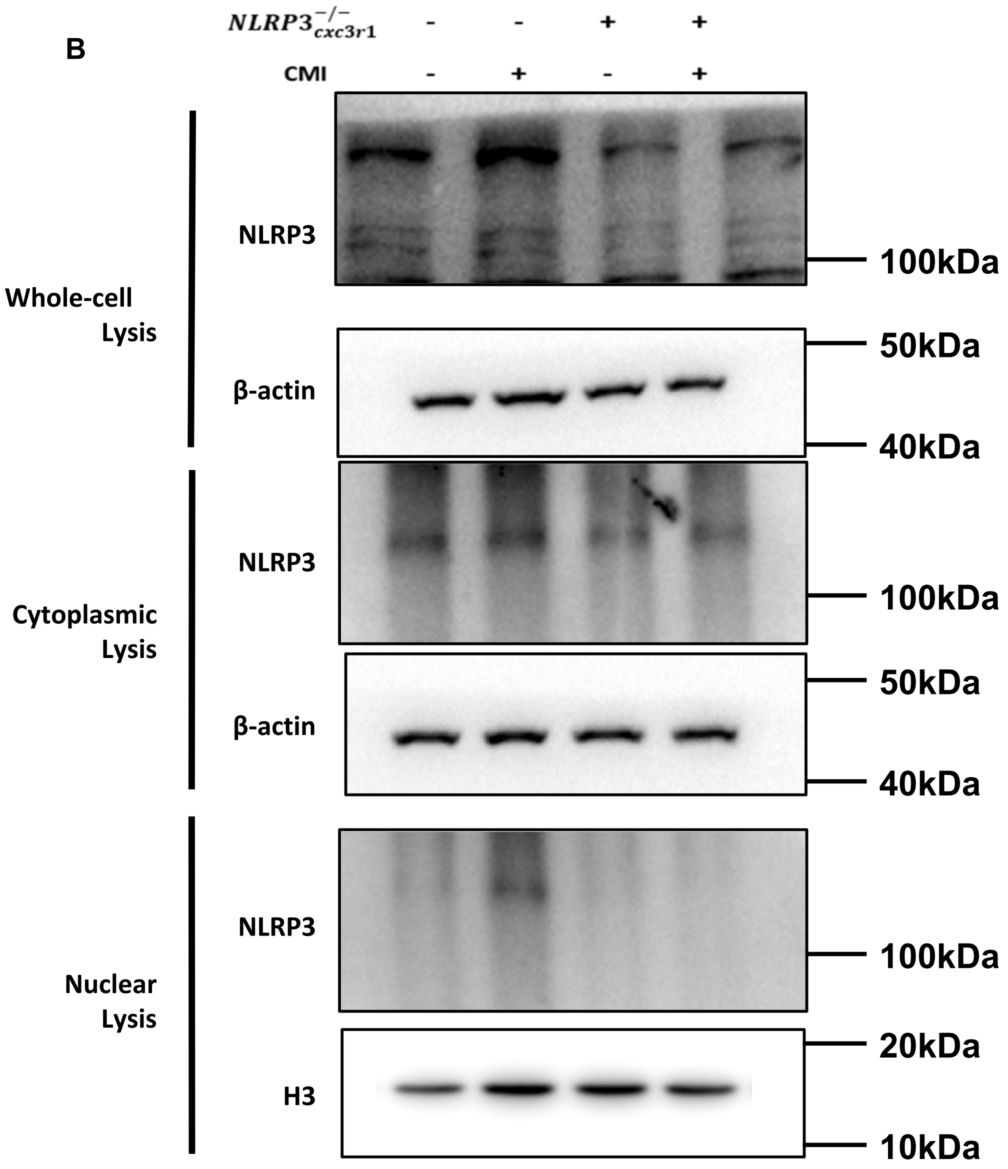

Full and uncropped western blot for Figure 5C

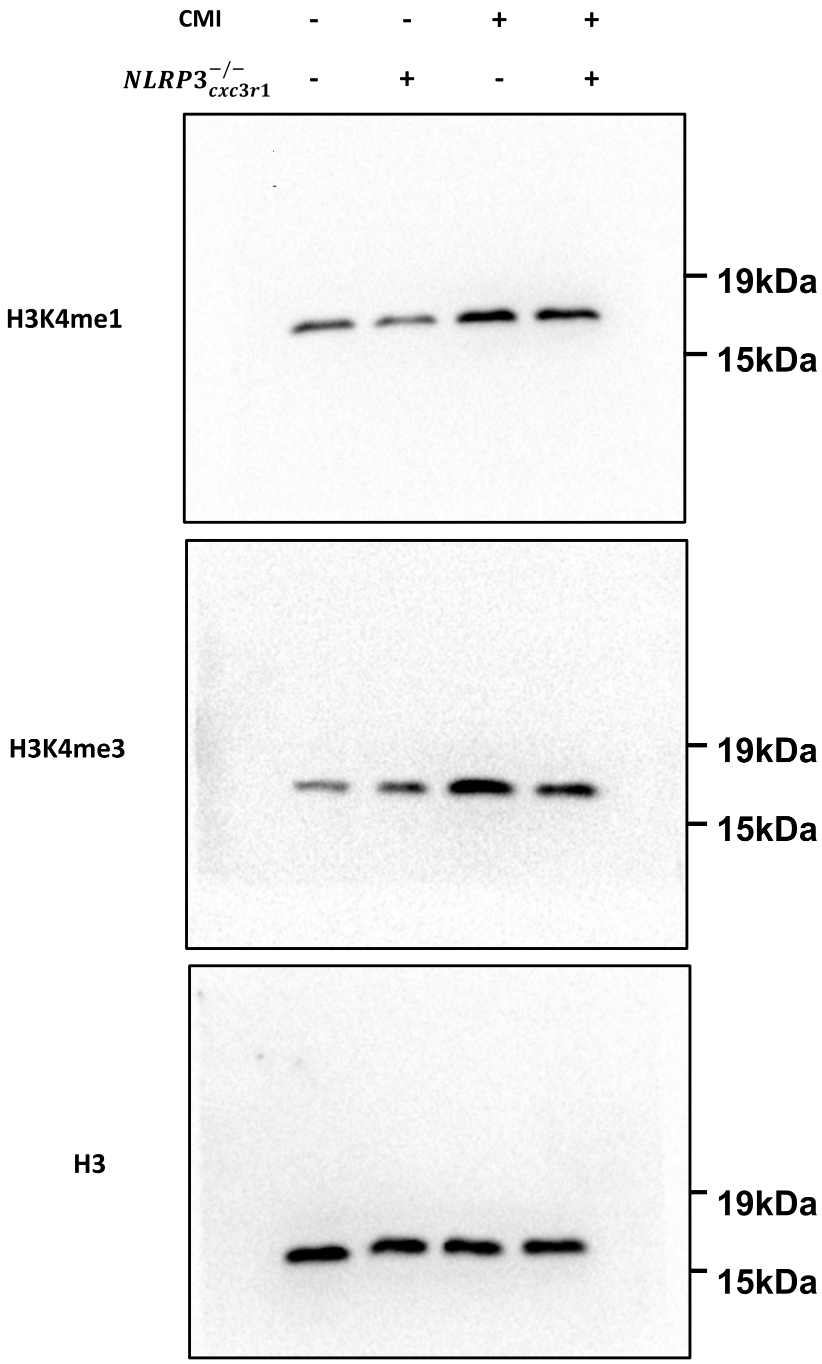

Full and uncropped western blot for Figure S1G

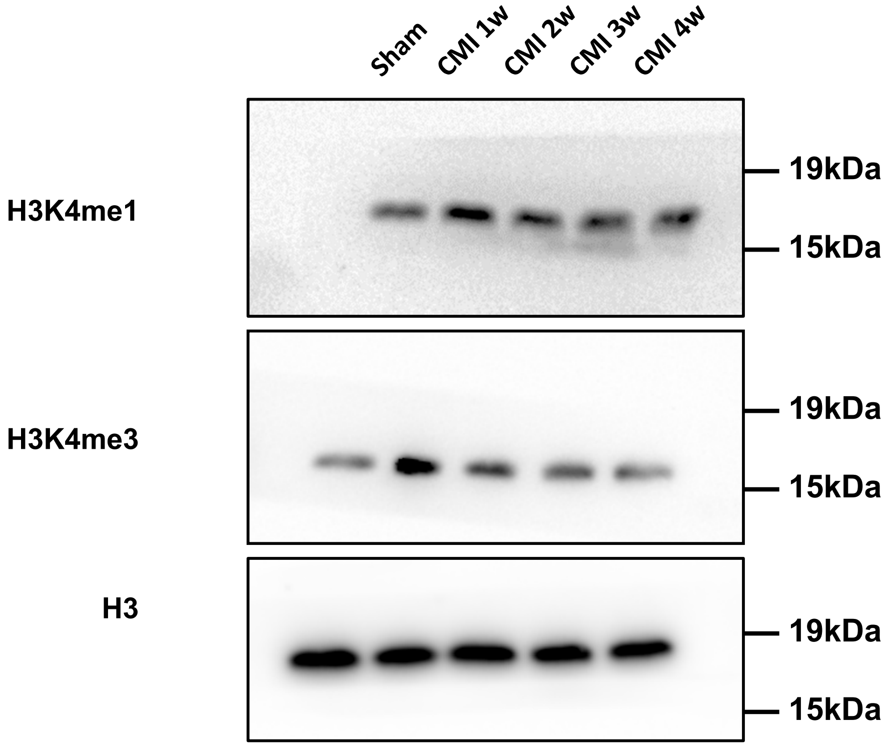

Full and uncropped western blot for Figure S2B

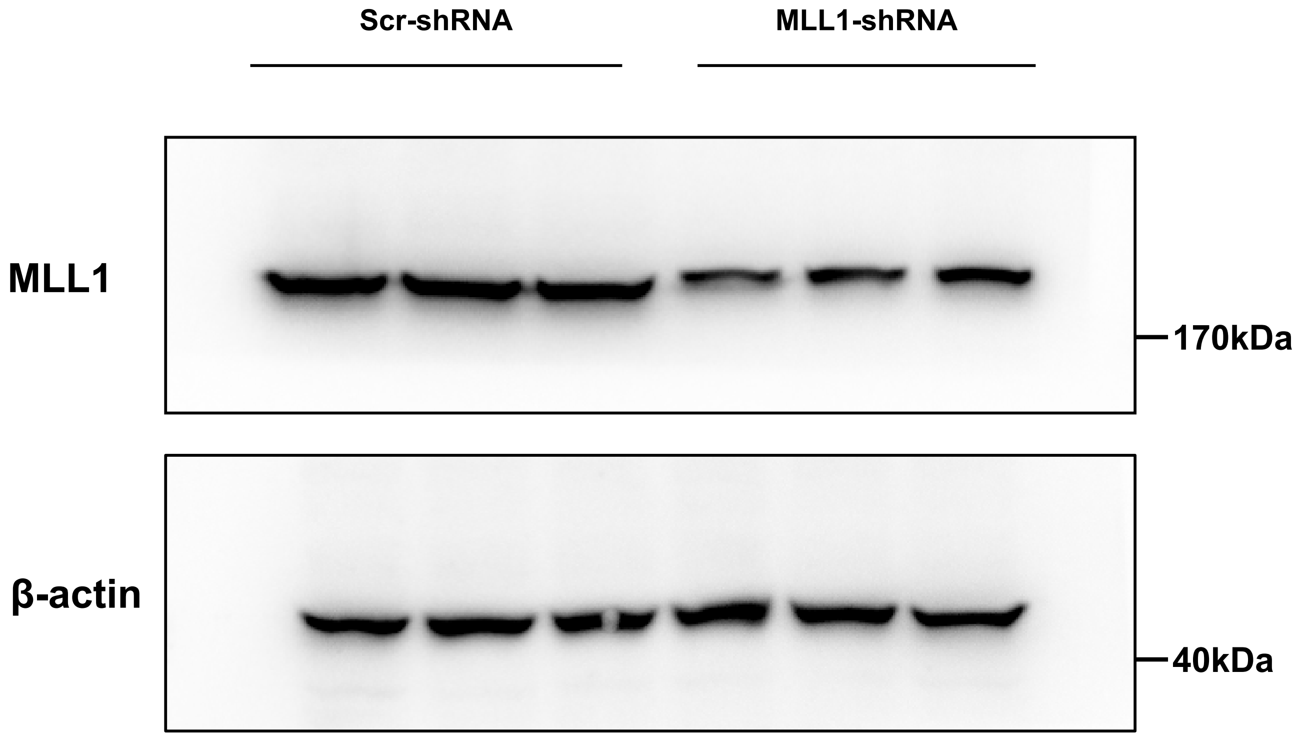

Full and uncropped western blot for Figure S4B

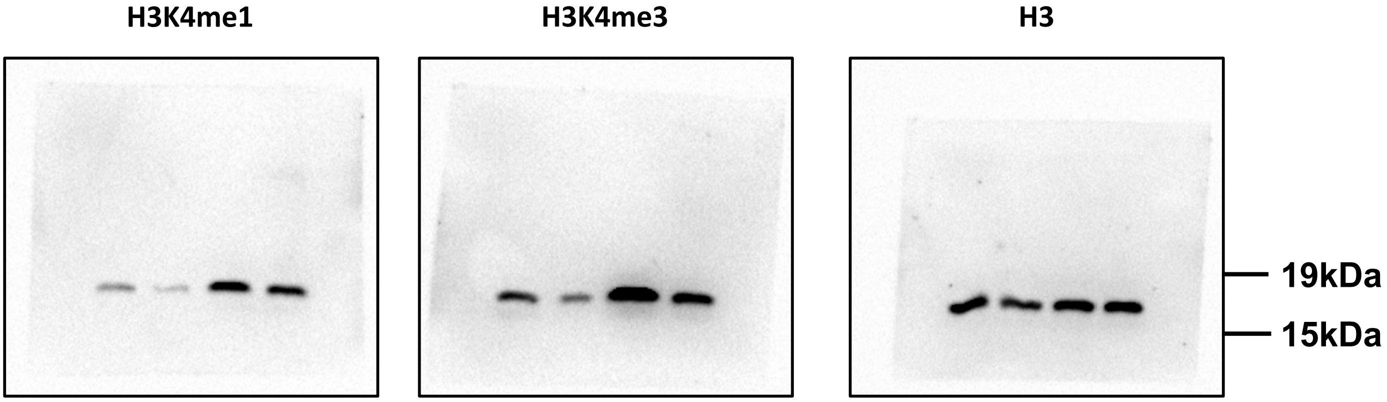

Full and uncropped western blot for Figure S4I

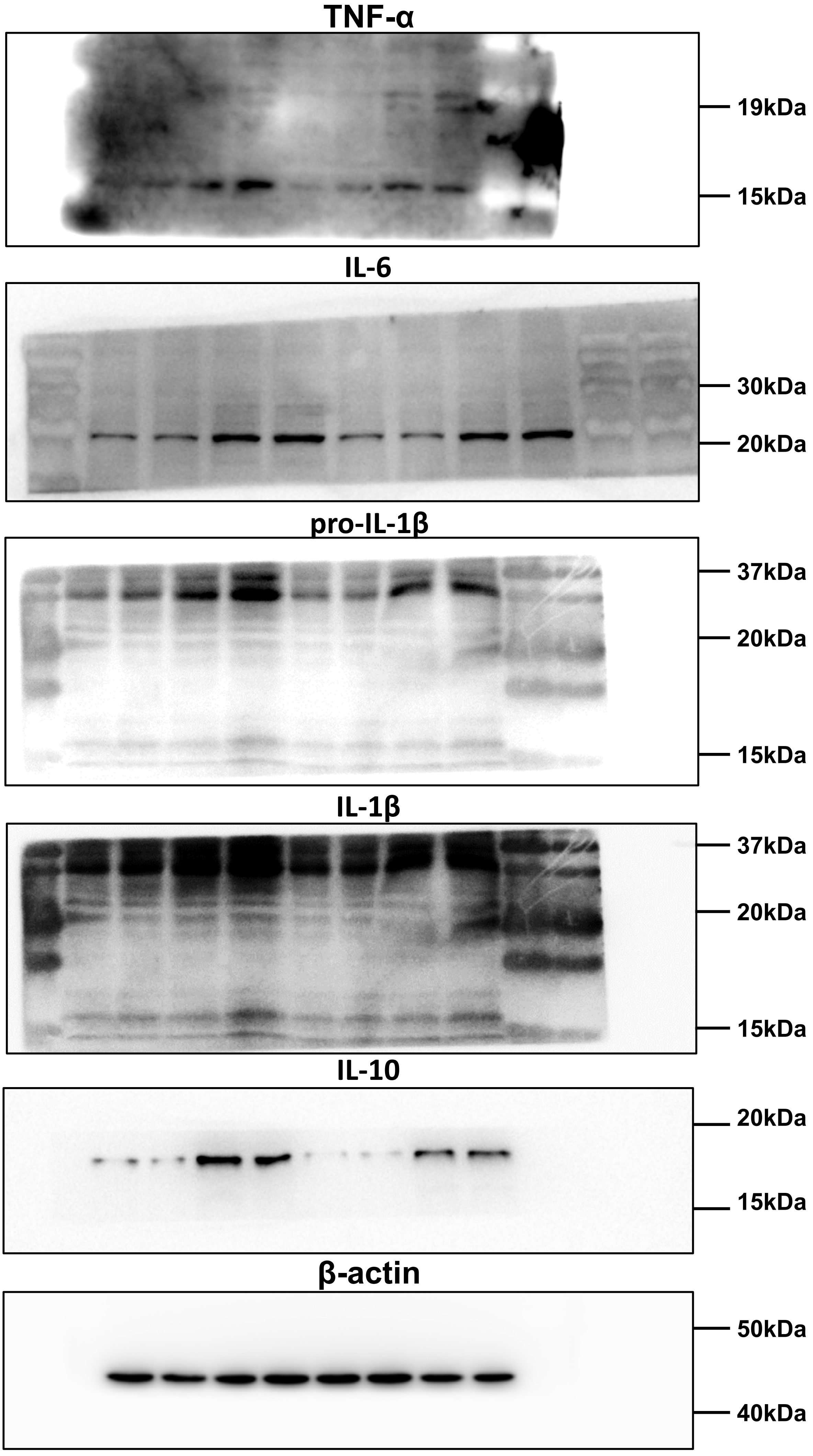

Supplement: Supplementary file 3 — Suplemantery material---WB [file 41419_2023_6414_MOESM3_ESM.pdf]
